# Parallel arrangements of positive feedback loops limit cell-to-cell variability in differentiation

**DOI:** 10.1371/journal.pone.0188623

**Published:** 2017-11-29

**Authors:** Anupam Dey, Debashis Barik

**Affiliations:** School of Chemistry, University of Hyderabad, Central University P.O., Hyderabad, Telangana, India; Universitat Pompeu Fabra, SPAIN

## Abstract

Cellular differentiations are often regulated by bistable switches resulting from specific arrangements of multiple positive feedback loops (PFL) fused to one another. Although bistability generates digital responses at the cellular level, stochasticity in chemical reactions causes population heterogeneity in terms of its differentiated states. We hypothesized that the specific arrangements of PFLs may have evolved to minimize the cellular heterogeneity in differentiation. In order to test this we investigated variability in cellular differentiation controlled either by parallel or serial arrangements of multiple PFLs having similar average properties under extrinsic and intrinsic noises. We find that motifs with PFLs fused in parallel to one another around a central regulator are less susceptible to noise as compared to the motifs with PFLs arranged serially. Our calculations suggest that the increased resistance to noise in parallel motifs originate from the less sensitivity of bifurcation points to the extrinsic noise. Whereas estimation of mean residence times indicate that stable branches of bifurcations are robust to intrinsic noise in parallel motifs as compared to serial motifs. Model conclusions are consistent both in AND- and OR-gate input signal configurations and also with two different modeling strategies. Our investigations provide some insight into recent findings that differentiation of preadipocyte to mature adipocyte is controlled by network of parallel PFLs.

## Introduction

Cellular heterogeneity is a natural phenomenon where an isogenic population of cells in homogeneous environmental conditions generate significant variability in cellular content, shape, size, cell cycle duration and stimuli responses[1, 2]. The origin of such heterogeneity has been found to be primarily due to the inevitable intrinsic[3–5] and extrinsic[6–11] sources of variabilities collectively known as chemical noise. While intrinsic noise originating from the fluctuations of low copy numbers of various chemical species is inherent to a chemical reaction, extrinsic noise globally influences all the chemical reactions in a cell. The chemical noise can either cause a nuisance in various cellular phenomena such as cell cycle[12], apoptosis[13], p53 dynamics[14], HIV virus latency/replication[15, 16], aneuploidy[17] or it can help cell efficiently adapt to the continuously changing environment[18, 19]. It has been a standing question how a living cell manages to minimize the effects of chemical noise which may have unwanted consequences in cellular phenomena.

Since the early findings that a negative feedback loop may attenuate the effects of stochastic fluctuations[20], a number of studies, both theoretical/computational[3, 21–26] and experimental[27–29], focused investigating on the role of negative feedback on the cellular noise. Negative feedback loop has the potential to reduce noise but at the cost of compromising the sensitivity to the external signals[30, 31]. On the other hand, calculations on simple network motifs by Hornung et al predicted that positive feedback has the ability to filter noise without compromising signal sensitivity[31]. In addition against a graded input signal a positive feedback loop (PFL) has potential to generate digital signal response creating bistability[32]. Bistability has been found to be associated with various cellular responses such as differentiation[33–35], memory[36, 37], activation of anaphase promoting complex in frog eggs[38, 39], G1/S transition in yeast and mammalian cells[40–42]. Consistent with experimental observations[41, 42], system-level stochastic model of budding yeast cell cycle[43] investigated the role of PFLs in various phases of the cell cycle and confirmed that PFL filters noise in various events during cell cycle.

Bistable switches are known to regulate cellular differentiations such as TGF-β induced epithelial to mesenchymal transition[33], preadipocyte to adipocyte differentiation[35] and myogenic and osteogenic differentiation[44]. In this context in order to lock a cell to its differentiated state, it must have tools to reduce the effects of noise such that the cell does not revert back to its original state and vice versa. This is quite relevant in situations where from a large pool of precursor cells only a small fraction differentiates under weak signaling regime. Fusing a negative feedback loop in bistable switch is known to increase the excitability of the system thus certainly it cannot be an optimal solution for reducing noise in bistable systems[45, 46]. Several protein regulatory networks are known to consist of two PFLs fused together[47–49] and model calculations showed that reliability of a noisy input signal increases if the two PFLs act in disparate time scales[50, 51]. Thus fusion of two or more PFLs can be a potential solution to this problem. However arrangements of these fused PFLs can have effect on the propagation of noise in the system. In a recent study Ahrends et al found that differentiation of preadipocyte to adipocyte cells located in fat tissue is regulated in a bistable manner created by seven independent positive feedback loops via a common master regulator peroxisome proliferator-activated receptor γ (PPARG) [52]. This independent arrangement of PFLs around the key regulator PPARG can be termed as parallel PFLs. Using model calculations they showed that added feedback loops efficiently reduces extrinsic noise as compared to a single feedback system with increased cooperativity.

However, the question that needs to be answered here is how different types of topologies of multiple PFLs dictate the noise in bistable systems. In particular, in addition to the parallel arrangements, PFLs can be arranged in a sequential manner creating a chain like topology that we call serial PFLs. Thus parallel and serial PFLs are analogous to parallel and serial arrangements of resistors in electrical circuits, respectively. The average properties, region of bistability and values of steady states, of parallel and serial circuits of PFLs can be exactly identical. However in presence of noise the behavior of these two topologies can be quite different and therefore can have detrimental effects in differentiation dynamics. Using mathematical modeling and stochastic simulations, here we investigated the effects of intrinsic and extrinsic noise in parallel and serial network motifs creating identical bistable switches. We found that, contrary to the serial motifs, a population of cell each with parallel PFLs is able to maintain their respective differentiation state in presence of either intrinsic or extrinsic fluctuations. Thus parallel PFLs are far superior in reducing the effects of noise compared to the PFLs with serial arrangement. Our calculations suggest that in parallel motifs the saddle-node bifurcation points generating bistability are less susceptible to extrinsic noise and the distributions of bifurcation points are less skewed as compared to serial motifs. Further, we found that the stability of steady states, calculated by mean residence times, are consistently higher in parallel motifs suggesting parallel motifs are less perturbed by intrinsic noise as compared to serial motifs. We found our results are consistent for both AND- or OR-gate configurations of input signals and are independent of modeling methodologies.

## Results and discussion

**Fig 1:**
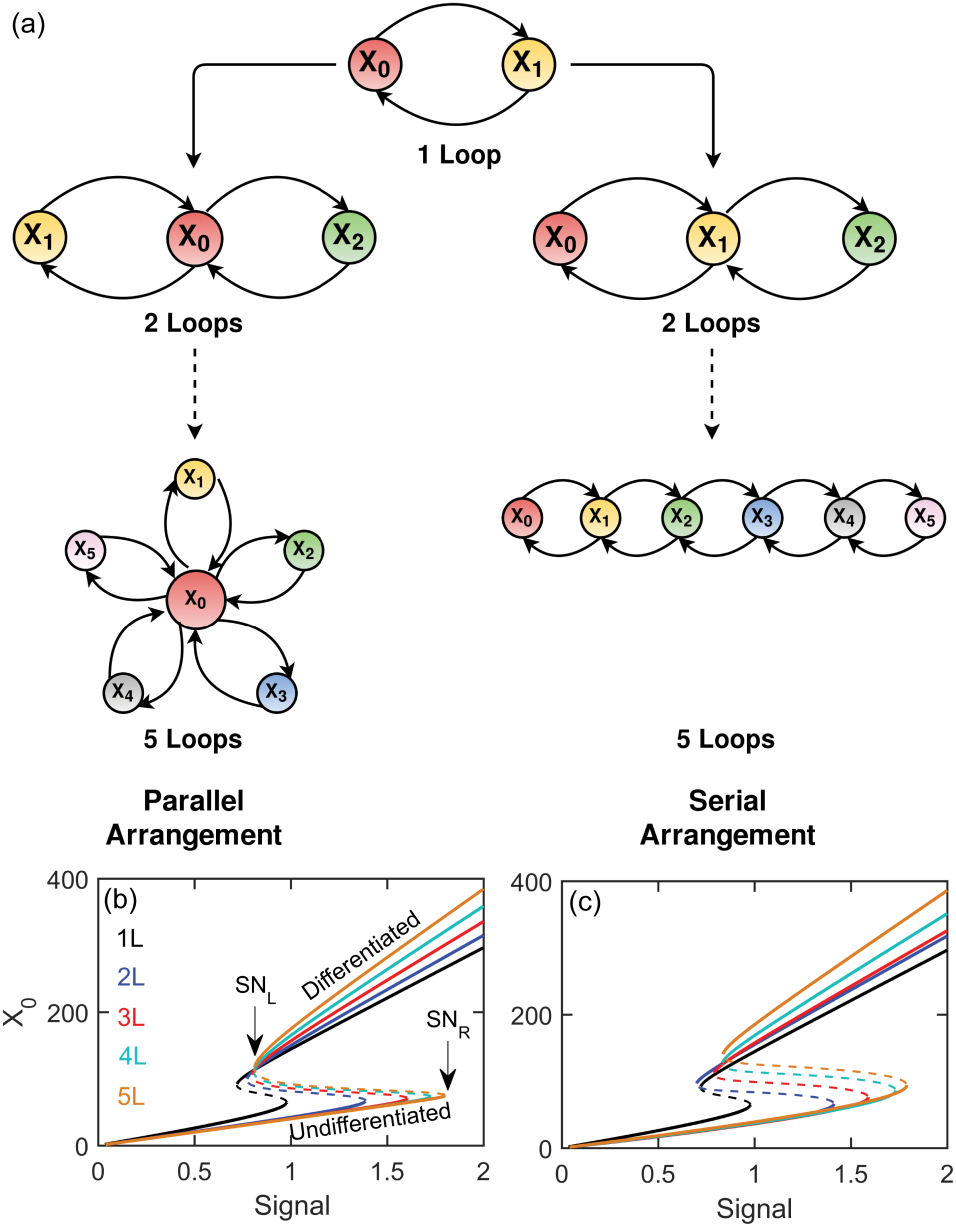
Network diagrams and bifurcations of models. (**a**) Positive feedback loop between two components (X_0_ and X_1_) creates a 1L PFL motif and several of these motifs are fused together to create either parallel (left) or serial motifs (right). One parameter bifurcation diagrams of parallel (**b**) and serial (**c**) models with various number of feedback loops with AND-gate configurations. The parameter *s* in the model equation represents the ‘signal’. The left and right saddle-node (SN_L_ and SN_R_) bifurcation points are indicated by the arrow. Upper and lower stable branches (solid line) are associated with the differentiated and undifferentiated states of a cell. The unstable middle branch (dashed line) separates these two states.

We investigated two classes of network motifs that generate bistable signal responses: *parallel* and *serial* PFLs. In *parallel* network motifs multiple PFLs are fused around a central regulator X_0_ creating a topology where feedback loops are independent of one another. On the other hand in *serial* network motifs PFLs are arranged serially in an end-to-end chain like sequence configuration (Fig 1). We first created a positive feedback loop between two components, X_0_ and X_1_, where they positively regulate synthesis of each other. This creates a single PFL motif (1L motif) and functions as a ‘repeat unit’ for multiple PFL motifs. Now to generate a parallel motif, for example a two-loop (2L) parallel motif, we introduced another component, X_2_, that is in a PFL with X_0_ independent of X_1_. In this way we generated networks with up to five parallel PFLs (5L) (Fig 1a, **left**). For the serial arrangements, the PFLs are interconnected with one another, for example in 2L serial motif, X_2_ is in PFL with X_1_ which is connected to X_0_ by a PFL (Fig 1a, **right**).

In signal transduction pathway often multiple input signals can act in non-redundant or redundant manner to trigger responses. Non-redundant input signals function like an AND-gate whereas redundant input signals function like an OR-gate analogous to electrical circuits[53]. We have taken into consideration of AND- or OR-gate configurations of signaling inputs in our models whenever applicable. For example, in parallel motifs synthesis of X_0_ is positively regulated by several components (X_1_, X_2_ etc.) either by AND- or OR-gate. Similarly in serial loops synthesis of any component X_i_ is regulated by X_i-1_ and X_i+1_ creating either AND- or OR-gate configurations except for the terminal components.

In a basic ‘repeat unit’ the synthesis rate of X_0_ is directly proportional to the amount of X_1_ present and in turn X_0_ helps the synthesis of X_1_ by enzymatically activating the transcription factor (T_1_) for X_1_. The production rate of X_1_ is proportional to the amount of its active transcription factor (T_1,A_). This single PFL motif (1L) may generate bistability satisfying the condition of embedded ultrasensitivity in the network[54]. In order to incorporate ultrasensitivity we implemented Goldbeter-Koshland’s (GK) zero order ultrasensitivity in the activation-deactivation of transcription factor[55]. As per the requirement of GK switch, we assumed that the total amount of transcription factor (*T*_*T*_) for every species is constant and activation-deactivation reactions follow Michaelis-Menten type enzyme kinetics. Owing to its flexibility in parameter space, Hill function has been a preferred choice to generate ultrasensitivity over Goldbeter-Koshland switch. However we chose the GK switch for accurate estimation of intrinsic noise using Gilespie’s algorithm[56, 57]. In the parallel arrangements for any component X_i_, its transcription factor T_i_ is activated enzymatically by X_0_ and in return all X_i_s catalyze the synthesis of X_0_ through either an AND- or an OR-gate input signaling combination. In case of serial arrangements, for any component X_i_, its transcription factor is activated by two neighboring components X_i-1_ and X_i+1_ by AND- or OR-gate mechanism. See S1 Fig for detailed network diagrams of serial and parallel models. We have listed the model equations in the Table 1 and provided the parameters corresponding to the models in the S1 and S3 Tables.

**Table 1:** List of dynamical equations for the parallel and serial models with GK switch.

Parallel^a^	Serial^b^
$\frac{dX_{0}}{dt}=s(k_{0}V+k_{1}P_{0})-\gamma X_{0}$	$\frac{dX_{0}}{dt}=s(r_{0}V+r_{1}X_{1})-\gamma X_{0}$
$\frac{dX_{i}}{dt}=k_{2}V+k_{2}^{\prime }T_{i,A}-\gamma X_{i}$	$\frac{dX_{i}}{dt}=r_{2}V+r_{2}^{\prime }T_{i,A}-\gamma X_{i}$
$\frac{dT_{i,A}}{dt}=\frac{k_{f}X_{0}(V.T_{T}-T_{i,A})}{K_{M}V+(V.T_{T}-T_{i,A})}-\frac{k_{b}V.T_{i,A}}{K_{M}V+T_{i,A}}$	$\frac{dT_{i,A}}{dt}=\frac{r_{f,i}G_{i}(V.T_{T}-T_{i,A})}{K_{M}V+(V.T_{T}-T_{i,A})}-\frac{r_{b}V.T_{i,A}}{K_{M}V+T_{i,A}}$

For *i* = 1,2, … *N*, where *N* = number of loops, *V* is a scaling factor to change the number of molecules of chemical species. The value of scaling factor (*V*) was 40 in all calculations.

^a^For AND-gate $P_{0}=\frac{1}{V^{N-1}}\prod_{i=1}^{N}X_{i}$ and for OR-gate $P_{0}=\sum_{i=1}^{N}X_{i}$.

^b^For AND-gate $G_{i}={\frac{1}{V}X}_{i-1}X_{i+1}$ and for OR-gate $G_{i}=X_{i-1}{+X}_{i+1}$; for *N* = 1, $G_{1}=X_{0}$ and for *i* = *N*, $G_{N}=X_{N-1}$.

In our models the synthesis rates of regulators (X_0_ and X_i_ s) consist of unregulated and regulated parts that follow mass action rate laws. The degradation rate constant (*γ*) or the life-time (1/*γ*) of all the components were assumed to be same representing their dilution dynamics during the growth of the host cell. We have used the same set of parameters for all motifs with parallel PFLs. Whereas, in case of serial arrangements the activation rate constant of transcription factor for X_1_ (*r*_*f*,*1*_)and the regulated synthesis rate constant of X_0_ (*r*_*1*_) were carefully adjusted to achieve similar region of bistabilities as in the case of parallel arrangements. In our models we have introduced a ‘cell volume’ parameter, *V*, to scale up or scale down the number of molecules of various species without hampering the dynamics of the model. Next we report the results from the models with AND configurations.

First we investigated the steady state responses of these networks in absence of any intrinsic or extrinsic sources of variabilities using XPP-AUT software tool[58] (http://www.math.pitt.edu/~bard/xpp/xpp.html) to generate one-parameter bifurcation diagrams of the models. These models generate reversible bistable switches while we varied the parameter *s* in the model. The parameter *s* represents the amount of external stimulus that triggers the differentiation of preadipocyte cells. The choice of parameter *s* as the bifurcation parameter is obvious as differentiation is typically triggered by extracellular signals, for example, in case of preadipocyte differentiation external stimulus rosiglitazone initiate adipogenesis in mouse OP9 cells[35]. We call this parameter as ‘signal’ in the rest of the paper. The parameter *k*_*0*_ or *r*_*0*_ represents the ‘effectiveness’ of the external signal in initiating differentiation. The bistability is due to the two saddle-node (SN) bifurcation points (Fig 1b) that create reversible bistable switches. With the increase in the number of feedback loops the region of bistability increases both in the parallel (Fig 1b) and serial (Fig 1c) topologies. We emphasize here that the bifurcation diagrams obtained from parallel and serial models are closely similar including the region of bistabilities. This is an important criteria for comparison of noise propagation in parallel and serial feedback loops. In the context of cell differentiation the lower and upper branches of stable steady states represent the undifferentiated (or dedifferentiated) and differentiated cellular states, respectively. The cell fate decision driven by SN bifurcation has been proposed in many systems [33, 59] although it is slightly different than the well celebrated Waddington’s epigenetic landscape[60] of cell fate decision. In the Waddington’s proposal of cell fate decision differentiation occurs due to the supercritical pitchfork bifurcation where in differentiation of preadipocyte cells SN bifurcations dictate the process. Several important consequences emerge due to the different nature of bifurcations regulating cell fate decision[59]. In case of SN bifurcation the alternate states are already present well before the critical point whereas in pitchfork bifurcation the new states are born only after the critical point. This has significant implications when the decision making processes are influenced by the molecular noise—before the critical point both the states can coexist in a population of cell in first case whereas in Waddington’s approach before critical point there is no possibility of coexistence of cell fates. Further cell fate decision making is always a reversible process in Waddington’s approach whereas cell differentiation driven by SN bifurcation can be irreversible.

In absence of any variability whatsoever every cell in a population would behave identically therefore there would be a clear switch-like transition from undifferentiated to differentiated state for the entire population when the signal dose crosses the right SN bifurcation point (SN_R_) (Fig 1b). Similarly the whole population of cells would shift sharply from the differentiated to the dedifferentiated (or undifferentiated) state when the signal is reduced beyond the left SN bifurcation point (SN_L_). Thus the population will be ‘pure’ in terms of the state of its differentiation in absence of any noise–they all are either differentiated or undifferentiated. However due to the extrinsic and intrinsic sources of variabilities each cell would behave differently resulting in a non-switch like response at the population level and ultimately this would generate a mixed population of differentiated and dedifferentiated cells in the intermediate range of signaling. To assess the extent of mixed population due to extrinsic and intrinsic noise, we calculated the percentage of differentiated (high X_0_, upper steady state) or dedifferentiated (low X_0_, lower steady state) cells with varying doses of signal (*s*).

**Fig 2:**
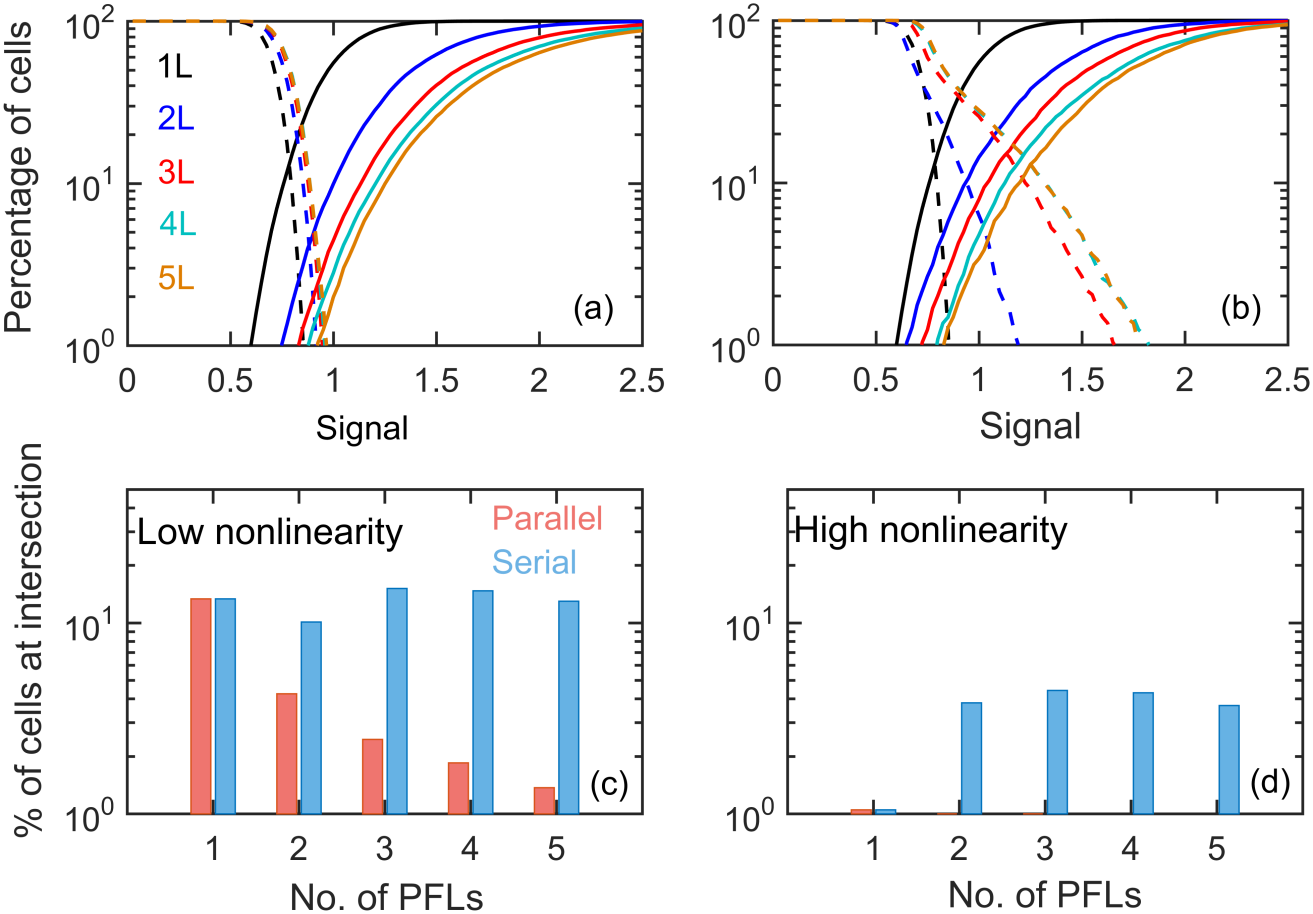
Differentiation with AND-gate under extrinsic noise. (**a**-**b**) The percentage of differentiated (solid) and dedifferentiated (dashed) cells with varying signal doses for parallel (left) and serial (right) motifs with different numbers of PFLs having low nonlinearity (*K*_*M*_ = 0.05). The percentage of cells at the intersection of differentiation and dedifferentiation curves with the number of PFLs are presented for low (**c**; *K*_*M*_ = 0.05) and high (**d**; *K*_*M*_ = 0.01) nonlinearity in both the models.

We implemented extrinsic noise in our models assuming that unregulated synthesis rates are log-normally distributed with a coefficient of variation (CV) of 30%. The choice of 30% variation in rate constants was due to the fact that similar variations have been observed[9] in many constitutively expressed proteins. Our objective here was to find out between the parallel and serial feedback loops which configuration filters noise more efficiently. We estimated the fraction of cell that transitions from undifferentiated (or differentiated) to differentiated (or dedifferentiated) state while the signal is varied in presence of extrinsic variability (see Materials and methods for details). We have performed separate calculations to estimate the fraction of differentiated and dedifferentiated cells with varying signal. In differentiation fraction calculation we started with undifferentiated state (initializing the system in the lower steady state of bifurcation diagram, low X_0_) and estimated the fraction of cells that have differentiated with varying signal. The sum of differentiated fraction and undifferentiated fraction (the population that did not differentiate) adds to 1. However we have reported only the differentiated fraction as our goal was to determine the effect of molecular noise on the differentiation starting from undifferentiated state. Similarly for the calculation of dedifferentiated fraction starting from differentiated state (initializing the system in upper steady state, high X_0_) we reported only the fraction of dedifferentiated cells. At any signal, the total fraction of differentiated and dedifferentiated cells does not add to 1 as we estimated these two populations from two separate calculations. Our aim here was to determine the fraction of cells that would differentiate at a given signal and once differentiated the fraction of cells that would dedifferentiate at the same signal in presence of extrinsic or intrinsic noise. Expectedly we found that with 1L motif, differentiation and dedifferentiation curves intersect with one another (Fig 2a and 2b) resulting in a mixed population in the intermediate range of signaling. In case of parallel motifs, as shown before[52], with the increase in number of feedback loops these two curves move away from one another minimizing the population heterogeneity in the low signal strength (Fig 2a). In serial arrangements, however, these two curves do not pull away from one another with increasing number of PFL resulting in a more heterogeneous population (Fig 2b). To estimate the extent of heterogeneity we calculated the percentage of cells at the intersection of these two curves. On the contrary to the serial motifs, the percentage at intersection decreases consistently with increasing numbers of feedback loops in parallel motifs (Fig 2c). Therefore serial motifs are less efficient in reducing the effect of extrinsic noise compared to the parallel PFLs although the underlying bistable switches are almost identical in both the cases. In the S2 Fig the steady state distribution of cells around the bifurcation diagram are given for parallel and serial motifs with extrinsic noise. In order to determine whether the noise suppression efficiency of parallel models depends on the nonlinearity or ultrasensitivity of the dynamics, we reduced the Michaelis constant in Goldbeter-Koshland switches (*K*_*M*_) by five times from 0.05 to 0.01. The decrease in Michaelis constant leads to increase in ultrasensitivity of the underlying switches. The bifurcation diagrams with increased value of nonlinearity are given in S3 Fig. We found that even with increasing nonlinearity the parallel PFLs are more efficient in reducing extrinsic noise as compared to serial motifs. However serial motifs performed slightly better with increasing nonlinearity (Fig 2d and S4 Fig).

**Fig 3:**
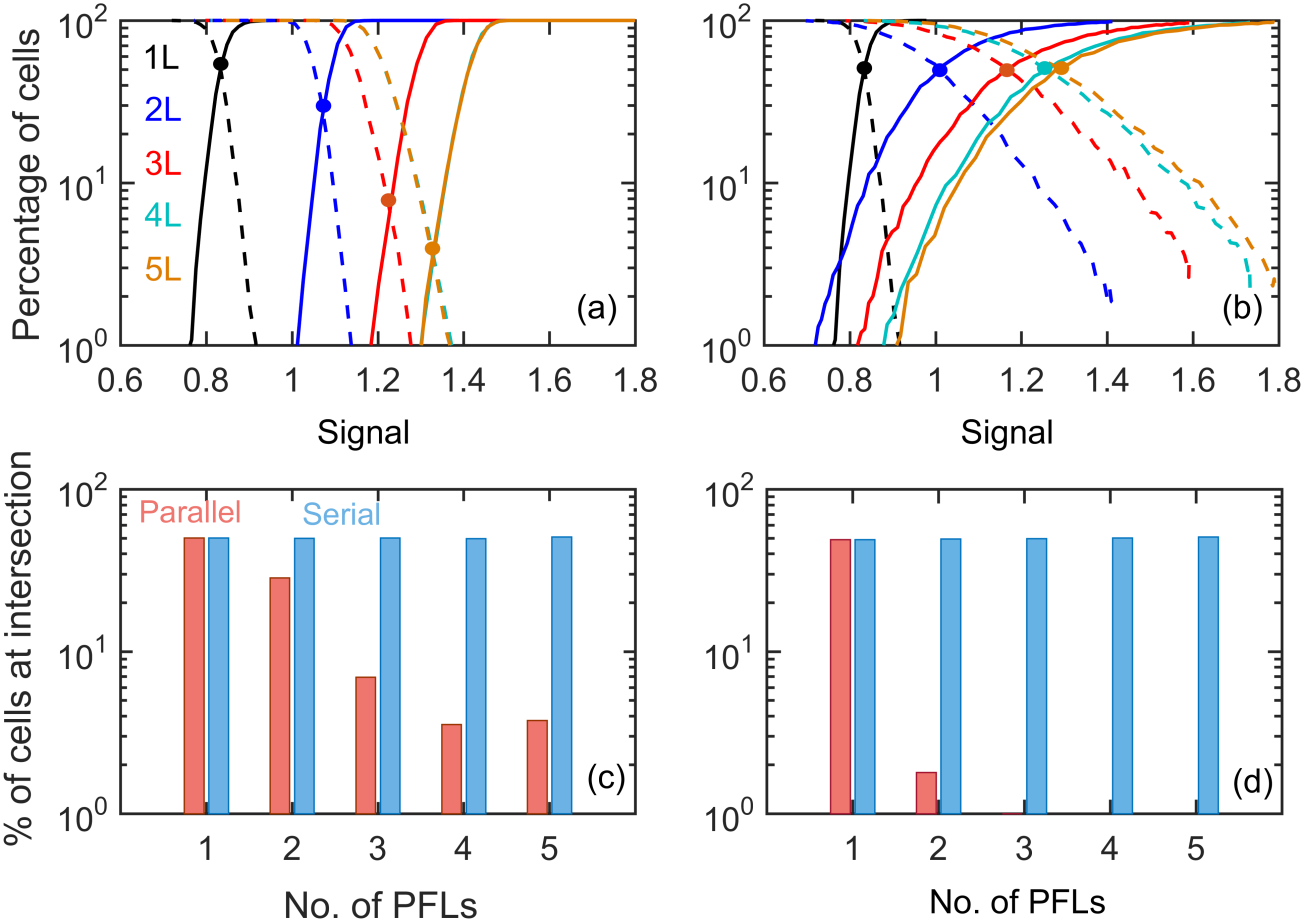
Differentiation with AND-gate under intrinsic noise. (**a**-**b**) The percentage of differentiated (solid) and dedifferentiated (dashed) cells with varying signal doses for parallel (left) and serial (right) regulatory motifs with different numbers of PFLs having low nonlinearity (*K*_*M*_ = 0.05). The percentage of cells at the intersection (indicated by solid circles in **a** and **b**) of differentiation and dedifferentiation curves with the number of PFLs are presented for low (**c**; *K*_*M*_ = 0.05) and high (**d**; *K*_*M*_ = 0.01) nonlinearity in Goldbeter-Koshland switches in both the models.

Intrinsic noise has been known to cause cell-to-cell variability through the ‘finite number effect’ in chemical reactions. Therefore we investigated the effects of intrinsic noise on the differentiation dynamics in both types of motifs by simulating the chemical reactions (S2 Table) of models using Gillespie’s stochastic simulation algorithm[56]. In the bistable region of bifurcation diagram the system (X_0_) may jump back-and-forth between the two stable steady states depending on the amount of noise the system possesses due to the stochasticity of molecular abundances. In the bistable region, the system alternates between the two stable steady states and at equilibrium some fraction of cells reside around the lower steady state and the rest reside around the upper steady states (S5 Fig). With increase in number of parallel PFLs the fraction of mixed population decreases (Fig 3a), whereas in serial case, even with multiple feedback loops there is no significant reduction of mixed population (Fig 3b). The percentage of mixed population at the intersection dramatically reduces with increasing number of feedback loops in parallel motifs contrary to the serial motifs (Fig 3c) the percentages are invariant to the number of feedback loops. Although the noise reduction capacity of parallel loops increases with the increase in nonlinearity, however there is no noticeable change in case of serial models (Fig 3d and S6 Fig). Therefore our calculations suggest that compared to serial arrangements of PFLs, parallel PFLs reduces both extrinsic and intrinsic noise more efficiently in bistable systems.

**Fig 4:**
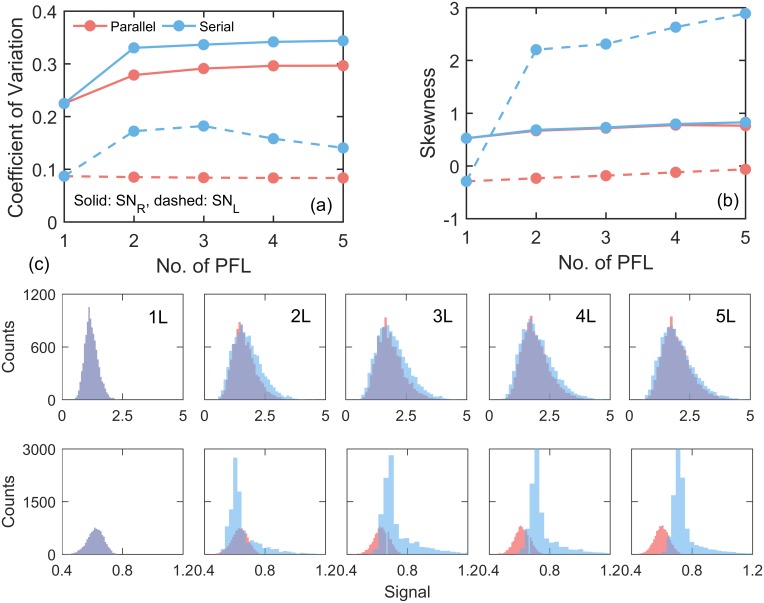
Susceptibility of bifurcation points to the extrinsic noise with low nonlinearity (*K*_*M*_ = 0.05) having AND-gate. The variation in coefficient of variation (**a**) and skewness (**b**) of distributions of right (SN_R_) and left (SN_L_) bifurcation points with increasing number of PFLs. (**c**) Comparison of the distributions of right (**c**; top row) and left bifurcation (**c**; bottom row) points for parallel and serial models.

We hypothesized that the sensitivity of saddle-node bifurcation points to the extrinsic noise must have significant role in dictating the noise in these two types of motifs. With extrinsic noise the bifurcation diagram of one cell will be different from another cell. Therefore we calculated the right and left bifurcation points (the value of signal, *s*) for 10000 cells in presence of extrinsic noise (Materials and methods). The amount of noise, CV, of SN bifurcation points in serial models are higher as compared to parallel models for both left and right bifurcation points (Fig 4a). We determined that higher noise in serial motifs was due to the increased skewness of distributions of bifurcation points (Fig 4b). While we found that the shape of distributions of right bifurcation points are similar for parallel and serial models, however the distributions of left bifurcation points are highly positively skewed for serial models as compared to the parallel models (Fig 4c). Consistently due to the increased skewness of left SN points the dedifferentiation curves had long tail in serial motifs (Fig 2b). Repeating calculations with decreased Michaelis constant we found similar results as well (S7 Fig). Thus the left bifurcation points, regulating the transition from differentiation to dedifferentiation state, are very much susceptible to extrinsic noise in serial PFLs.

**Fig 5:**
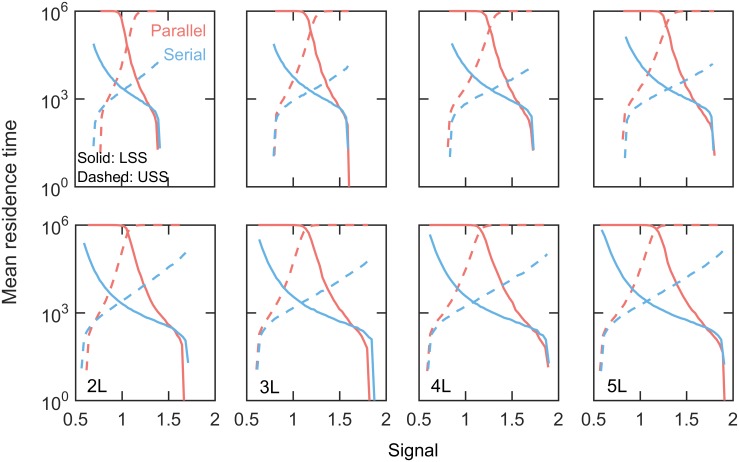
Stability of steady states under intrinsic noise: Mean residence time. Comparison of mean residence times of upper (USS) and lower (LSS) steady states for parallel and serial models with AND-gate. Top row: low nonlinearity (*K*_*M*_ = 0.05) and bottom row: high nonlinearity (*K*_*M*_ = 0.01). The maximum simulation time was 1×10^6^ arbitrary time unit.

The susceptibility of a steady state to the intrinsic noise is determined by the stability of the steady states[61]. Therefore to assess the stability of the steady states we calculated the mean residence time of steady states in the bistable region of the bifurcation diagrams (Materials and methods). The mean residence times of both upper and lower stable branches of bifurcations are much higher in parallel motifs as compared to the serial motifs (Fig 5). This clearly indicates that owing to the higher stability of steady states in parallel feedback loops, the system becomes less susceptible to stochastic fluctuations of molecular abundance or intrinsic noise. Thus PFLs with parallel arrangements efficiently are able to filter intrinsic noise. The increased sensitivity of steady states to the intrinsic noise in serial motifs is due to the chain-like architecture of PFLs where small amplitude fluctuations in abundance propagate and get amplified on its way to the activation of terminal regulator X_0_. On the other hand small fluctuations neither propagate nor amplified due to the independent architecture of PFLs in parallel motifs.

**Fig 6:**
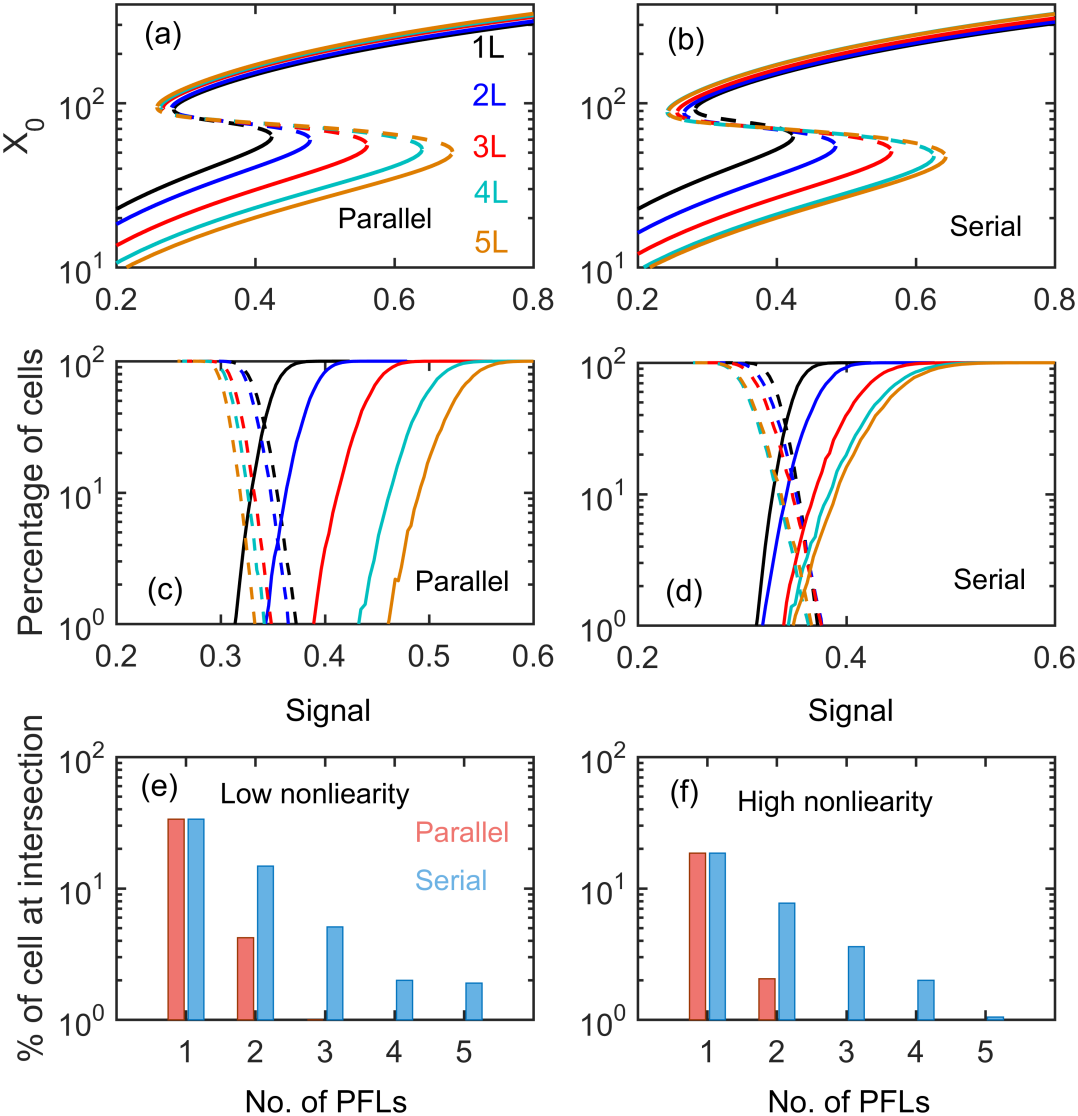
Differentiation in OR-gate configuration. One parameter diagrams (**a**-**b**), differentiation and dedifferentiation percentage (**c-d**) under intrinsic noise with low nonlinearity and the extent of mixed population with low (**e**) and high (**f**) nonlinearities in Goldbeter-Koshland switch.

We further extended our calculations in case of OR-gate configurations where signaling can trigger in redundant manner (for parameters see S3 Table). We performed similar calculations as we did for the AND case by parameterizing models such that both parallel and serial models generate similar bistable switches (Fig 6a and 6b). In OR-gate configurations parallel motifs efficiently reduce both extrinsic (S8 Fig) and intrinsic noise compared to the serial motifs although serial motifs does better job in OR as compared to AND case (Fig 6c–6f). From the mean residence time calculations, we also found that the stability of stable branches in parallel PFLs are higher than in the serial loops (S9 Fig). From these calculations we found that the OR-gate signaling input show less variability when compared to the AND-gate signaling input because fluctuations in OR-gate gets amplified due to the multiplicative nature of the gate.

## Materials and methods

### Calculation of differentiation-dedifferentiation percentage

We have calculated differentiation/dedifferentiation percentages for a population of cells in presence of extrinsic and intrinsic noise with varying signal doses. Single cell quantification of many proteins in eukaryotic cells revealed asymmetric and positively skewed protein distributions which can be best fitted by log-normal distributions [9, 13, 62]. Several extrinsic factors including differences in cell size, shape, cellular content, cell cycle phases, local temperature and pH are known to contribute significantly in population heterogeneity of protein abundance and ultimately leads to positive skewness in distributions. Therefore in order to generate log-normal distributions of proteins we have introduced log-normal distribution of rate constants which globally takes into account of various extrinsic factors together. We implemented extrinsic noise assuming log-normal distribution of unregulated synthesis rate constants (for parallel *k*_*0*_ and *k*_*2*_ and for serial *r*_*0*_ and *r*_*2*_) with 30% variation as unregulated proteins were found[9] to follow log-normal distribution with a typical CV of 30%. The average values of rate constants are listed in S1 Table. The typical sample size in our calculations was 10000 representing a sample of 10000 ‘cells’. It is also important to mention that for every component X_i_ its corresponding rate constant *k*_*2*_ or *r*_*2*_, as appropriate, was sampled from a different sequence of random numbers to generate independent distributions.

In order to calculate the percentage of cell differentiated at a given signal dose, *s*, we initialized the system in lower steady state of the bifurcation diagram and numerically solved the coupled set of ordinary differential equations listed in Table 1 using CVODE method (https://computation.llnl.gov/projects/sundials/cvode) implemented in Matlab. At each input signal *s*, we simulated a total of 10000 cells sampling the basal synthesis rate constants from log-normal distributions as mentioned. We integrated the set of equations for sufficiently long time to ensure that the system reached steady state. Finally to determine the number of differentiated cells, we counted the number of cells that have reached above a certain threshold value of X_0_ at steady state. For differentiation, the threshold was chosen as the value of X_0_ corresponding to the right saddle-node bifurcation point. In dedifferentiation fraction calculation, on the contrary, we initialized the system at the upper steady state and following the similar procedure we counted the number of cells below a threshold value of X_0_ corresponding to the left bifurcation point.

In case of intrinsic noise originating from the fluctuations of finite number of molecular species, we used Gillespie’s stochastic simulation algorithm [56] to simulate the reactions (S2 Table) corresponding to the dynamical equations listed in the Table 1. Here to determine the fraction of differentiated cells, we recorded the steady state value of X_0_ simulating the reactions for sufficiently long time after initializing the system at the lower steady state of the bifurcation diagram. At each value of input signal *s*, we generated a steady state distribution of X_0_ (S5 Fig). We counted the number of cells with values of *X*_0_ above the separatrix (unstable steady state) to calculate the fraction of differentiated cells. We performed similar calculations initializing the system from the upper steady state to calculate the percentage of dedifferentiated cells at steady state. Here we counted the number of cells that were present below the separatrix.

### Sensitivity of bifurcation points

Due to the extrinsic noise, the locations of saddle-node bifurcation points shift from one cell to another cell. We determined the two saddle-node bifurcation points for 10000 cells in presence of extrinsic noise to estimate the sensitivity of bifurcation point to the noise. For each cell, we integrated numerically the dynamical equations with the varying values of the signal *s*. The bifurcation point was the value of *s* for which the system abruptly jumps from one steady state to the other. By repeating this calculations for 10000 cells initializing the system either from lower or upper steady state, we generated two distributions corresponding to right and left saddle-node bifurcation points, respectively.

### Residence time calculations

In the bistable region of bifurcation diagrams, we calculated the mean residence time of steady states (undifferentiated and differentiated states) while the system is influenced by the intrinsic noise. We simulated the chemical reactions initializing the system at the lower steady state and recorded the time at which the system crosses the separatrix for the first time to get the first passage time or residence time of the lower steady state. By repeating this calculations 10000 times, we obtained the mean residence time for the lower steady state. We followed the same procedure to calculate mean residence time of differentiated state initializing the system at the upper steady state. The maximum time of calculations was 1×10^6^ arbitrary time unit.

## Conclusions

Cellular functions are regulated by chemical reaction networks with distinct steady state and dynamical properties. These properties help the regulatory system to achieve desired cellular functions. The properties of regulatory network motifs depend crucially on the architecture or the topology of these networks, for example, a positive feedback loop generates multistability or a negative feedback loop generates oscillations or excitability[63]. The average properties of topologically equivalent networks could be similar although the properties of these networks are known to get perturbed differently due to extrinsic and intrinsic sources of chemical noise. Since in many cases chemical noise is known to act as nuisance to the cellular behavior, therefore the system’s obvious tendency would be to adopt a network that helps minimize the effects of chemical noise. In this context, a positive feedback loop that generates bistability has been found to reduce fluctuations in various cellular phenomena[31, 41–43]. On the other hand bistability generated from fusion of multiple PFLs have been found to regulate many cellular differentiation processes[33, 35, 37]. Multiple PFLs help the system to generate a robust bistability, however the arrangements of these PFLs may have some crucial role in reducing the effects of chemical noise. Therefore the topological effects of multiple PFLs on the stochasticity of a network is quite relevant in order to understand how a cell lock the undifferentiated or differentiated state under the influence of chemical noise.

Recently Ahrends et al[52] showed that the differentiation to mature adipocyte from a large pool of preadipocyte cells are controlled by bistable switch and the low rate of differentiation is maintained by stochastic fluctuations of chemical species within the regime of weak signaling. However the same stochasticity may lead to the loss of differentiated state, thus locking a differentiated state is a crucial task a cell has to achieve. This indicates that there must be some mechanisms in place that can filter the effects of chemical noise in cellular differentiation. As argued before, the network topology may have some relevance in the noise filtration[47, 50, 51]. The most intriguing finding of their work was that the differentiation of preadipocyte cells is controlled by seven PFLs around a central regulator PPARG creating a parallel arrangements of PFLs. Thus a relevant question that arises here is why these PFLs are arranged in a parallel manner while a serial arrangements may as well serve the purpose.

In order to address the question how the network topologies of positive feedback loops contribute to the amplification or reduction of chemical noise, we generated bistable switches with similar region of bistabilities from parallel and serial arrangements of PFLs. We calculated the fraction or percentage of differentiated and de-differentiated cells both for parallel and serial topologies consisting of different numbers of feedback loops. We found that when the signals are in AND-gate configurations parallel topologies reduce both extrinsic and intrinsic noise more efficiently compared to the serial topologies with a given number of loops. In fact we found that parallel motifs are much efficient in filtering intrinsic noise as compared to the serial motifs. Our calculations indicate the two saddle-node bifurcation points leading to bistability are much sensitive to the extrinsic noise when the topologies are serial in nature. This increased sensitivity is reflected on the skewness of the distribution of bifurcation points in serial motifs and ultimately increases variability in differentiated state. On the other hand In case of intrinsic noise the stability of the steady states, measured by mean residence time, in the bistable region are much higher in parallel motifs compared to the serial motifs. Therefore steady states in parallel motifs are less susceptible to intrinsic noise as compared to serial motifs. We also investigated the OR-gate input signal configurations and found that the intrinsic noise filtering capacity of parallel motifs are again much better than serial motifs.

Further, in order to find out whether our conclusions are dependent on modeling methodology, we used Hill functions to model the ultrasensitivity instead of Goldbeter-Koshland switch. We performed similar calculations (for equations and parameters see S4 and S5 Tables) as we reported in the **Results and Discussion** section and found that with Hill functions parallel motifs reduce both extrinsic and intrinsic noise efficiently as well (S10 Fig).

In absence of any stochasticity parallel and serial motifs would not make any difference in differentiation dynamics. However our investigations of extrinsic and intrinsic noise in parallel and serial PFLs showed that parallel motifs reduce noise significantly better compared to serial motifs. Therefore evolution may have chosen parallel configurations as it is robust to the stochastic fluctuations of chemical species. As investigated previously[50, 64] that the complexity of PFLs have potential to reduce noise however our results also emphasize that in addition to the complexity the topological arrangements of PFLs play a major part in noise attenuation. Parallel architecture of positive feedback loops are not limited only to the differentiation. In cell cycle network of *Saccharomyces cerevisiae* (budding yeast), activation of b-type cyclins Clb1,2 are regulated by three positive feedback loops through independent involvement of Cdh1, Sic1 and Fkh2 in OR-gate configuration. [43]Stochastic model of cell cycle predicted that removal of any one of these positive feedback loops increases variability in various cell cycle properties such as cycle time, size at birth and division etc. Further similar architecture of PFLs are also known to present in activation of maturation promoting factor (MPF) in cell cycle network of *Saccharomyces pombe* (fission yeast)[65].

In our models the parameter values are within the realistic range of biological parameters. For proteins we have chosen ~70 min (ln(2)/*γ*) as the half-life which is typical average half-life of many proteins. The chosen synthesis rate constants lead to the molecular abundances in the range of a few hundred molecules per cell which also falls in the physiological range. We have performed simulations where rate constants were picked up from log-normal distributions (CV = 0.3) that takes into account of reasonable range in parameter values. Further we have studied two different configurations of input signals (AND- and OR-gate) and we also extended the modeling approach using Hill function to generate bistable switch. In all cases our calculations produced similar conclusions indicating the generality of our findings.

## Supporting information

S1 Codes Computer codes used to produce the data reported in the manuscript. (ZIP)

S1 Fig Detailed networks for 1L and 2L PFL motifs. Detailed networks for 1L PFL, 2L parallel (left) and 2L serial (right) motifs.
(TIFF)

S2 Fig The steady state distribution of cells in presence of extrinsic noise. The steady state distribution of cells in presence of extrinsic noise for various number of PFLs with low nonlinearity (*K*_*M*_ = 0.05) for the Goldbeter-Koshland switch model with AND-gate. Each point here represents a cell. The upper two rows (blue) and the lower two rows (orange) have cells initialized in the lower and upper steady states respectively.
(TIF)

S3 Fig One parameter bifurcation diagrams with high nonlinearity. One parameter bifurcation diagrams for parallel (left) and serial (right) models with AND-gate for various number of loops with high nonlinearity (*K*_*M*_ = 0.01) for the Goldbeter-Koshland switches.
(TIF)

S4 Fig Differentiation with extrinsic noise and high nonlinearity. Differentiation with extrinsic noise and with high nonlinearity (*K*_*M*_ = 0.01) for the Goldbeter-Koshland switch models with AND-gate. **(a-b)** The percentage of differentiated (solid) and dedifferentiated (dashed) cells with varying signal doses for parallel (left) and serial (right) regulatory motifs with different numbers of PFLs.
(TIF)

S5 Fig The steady state distribution of cells in presence of intrinsic noise. The steady state distribution of cells in the bistable region with the intrinsic noise for various number of PFLs with low nonlinearity (*K*_*M*_ = 0.05) for the Goldbeter-Koshland switches with AND-gate. Each point here represents a cell. The upper two rows (blue) and the lower two rows (orange) have cells initialized in the lower and upper steady states respectively.
(TIF)

S6 Fig Differentiation with intrinsic noise and high nonlinearity. Differentiation under intrinsic noise with high nonlinearity (*K*_*M*_ = 0.01) for the Goldbeter-Koshland switch models with AND-gate. (**a**-**b**) The percentage of differentiated (solid) and dedifferentiated (dashed) cells with varying signal doses for parallel (left) and serial (right) regulatory motifs with different numbers of PFLs are shown.
(TIF)

S7 Fig Susceptibility of bifurcation points to the extrinsic noise. Susceptibility of bifurcation points to the extrinsic noise for the Goldbeter-Koshland switch models with AND-gate having high nonlinearity (*K*_*M*_ = 0.01). The coefficient of variation (top left) and skewness (top right) of right (SN_R_) and left (SN_L_) bifurcation points with increasing number of PFLs are shown. Comparison of the distributions of right (top row) and left bifurcation (bottom row) points for parallel and serial models.
(TIF)

S8 Fig Differentiation with extrinsic noise in OR-gate models. Differentiation with extrinsic noise with low nonlinearity (*K*_*M*_ = 0.05) for the Goldbeter-Koshland switch models with OR-gate. The percentage of differentiated (solid) and dedifferentiated (dashed) cells with varying signal doses for parallel (left) and serial (right) regulatory motifs with different numbers of PFLs.
(TIF)

S9 Fig Mean residence time of steady states with OR-gate. Stability of steady states under intrinsic noise: mean residence time steady states with OR-gate. Comparison of mean residence times of upper (USS) and lower (LSS) steady states for parallel and serial models with low nonlinearity (top; *K*_*M*_ = 0.05) and High nonlinearity (bottom; *K*_*M*_ = 0.01). The maximum simulation time was 1×10^6^ arbitrary time unit.
(TIF)

S10 Fig Differentiation in AND-gate Hill function models with extrinsic and intrinsic noise. One parameter bifurcation diagrams **(a-b)** for parallel (left) and serial (right) models with Hill function with AND-gate for various number of loops with cooperativity (*M* = 2). The color scheme of lines are same as S8 Fig. Comparison of the percentage of cell at the intersection of differentiation and dedifferentiation curves for two models with various number of PFLs. **(c)** extrinsic noise and **(d)** intrinsic noise.
(TIF)

S1 Table Parameter values for the models in AND-gate configurations. Parameter values for the models in **AND**-gate configurations. With changing number of feedback loops, the parameters whose values were adjusted to obtain similar region bistability are highlighted. Red-coloured fonts indicate that in case of extrinsic noise calculations these rate constants were sampled from independent log-normal distributions (CV = 0.3) with average value indicated in the table. The unit of *r*_*f*,*i*_ becomes min^-1^ for *i* = *N*. The value of scaling factor (*V*) was 40.
(DOCX)

S2 Table List of chemical reactions and their propensities. List of chemical reactions and their propensities in parallel and serial models with GK switch.
(DOCX)

S3 Table Parameter values for the models in OR-gate configurations. Parameter values for the models in **OR**-gate configurations. With changing number of feedback loops, the parameters whose values were adjusted to obtain similar region bistability are highlighted. Red-coloured fonts indicate that in case of extrinsic noise calculations these rate constants were sampled from independent log-normal distributions (CV = 0.3) with average value indicated in the table. The value of scaling factor (*V*) was 40.
(DOCX)

S4 Table Dynamical equations for the models with Hill function. List of dynamical equations for models with Hill function.
(DOCX)

S5 Table Parameters values for the models with Hill function. Parameters values for the models with Hill function. Red-coloured fonts indicate that in case of extrinsic noise calculations these rate constants were sampled from independent log-normal distributions (CV = 0.3) with average value indicated in the table. The value of scaling factor (*V*) was 30.
(DOCX)
