# Supplementary figures and images for: Parallel arrangements of positive feedback loops limit cell-to-cell variability in differentiation
Source: PLoS One. 2017 Nov 29;12(11):e0188623. doi: 10.1371/journal.pone.0188623 (PMC5706692; doi:10.1371/journal.pone.0188623)

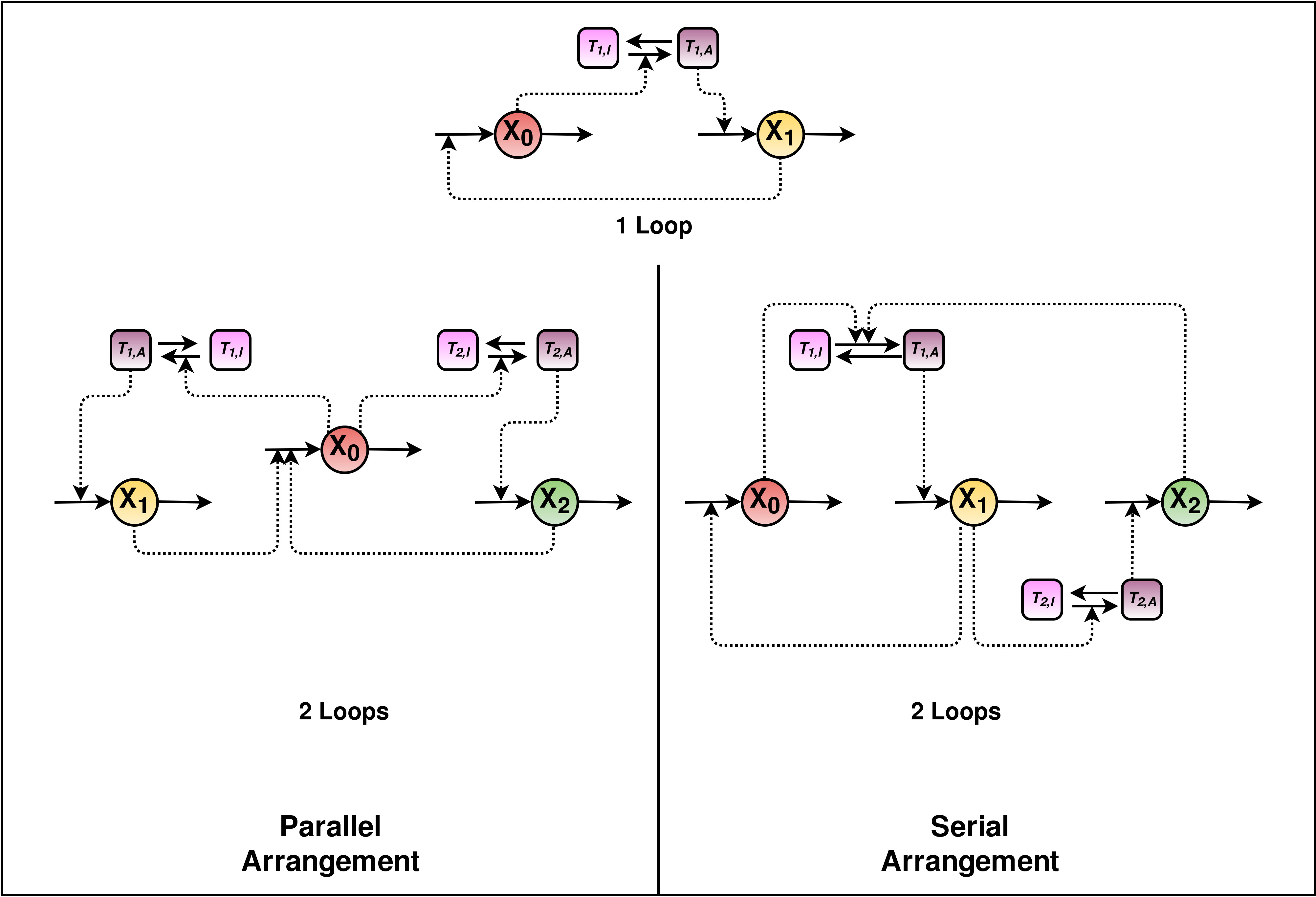

Supplement: S1 Fig — Detailed networks for 1L PFL, 2L parallel (left) and 2L serial (right) motifs. (TIFF) [file pone.0188623.s002.tiff]

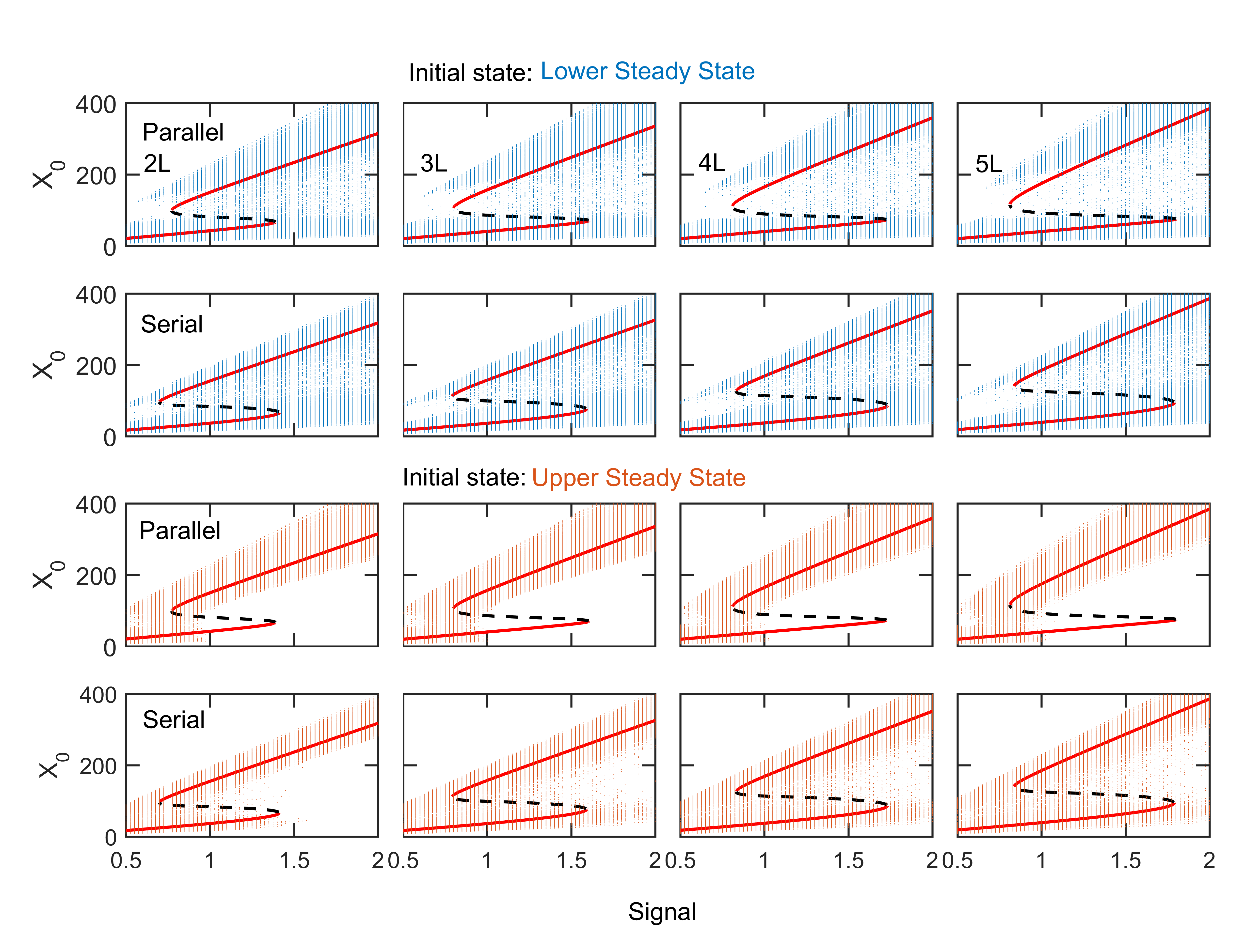

Supplement: S2 Fig — The steady state distribution of cells in presence of extrinsic noise for various number of PFLs with low nonlinearity (KM = 0.05) for the Goldbeter-Koshland switch model with AND-gate. Each point here represents a cell. The upper two rows (blue) and the lower two rows (orange) have cells initialized in the lower and upper steady states respectively. (TIF) [file pone.0188623.s003.tif]

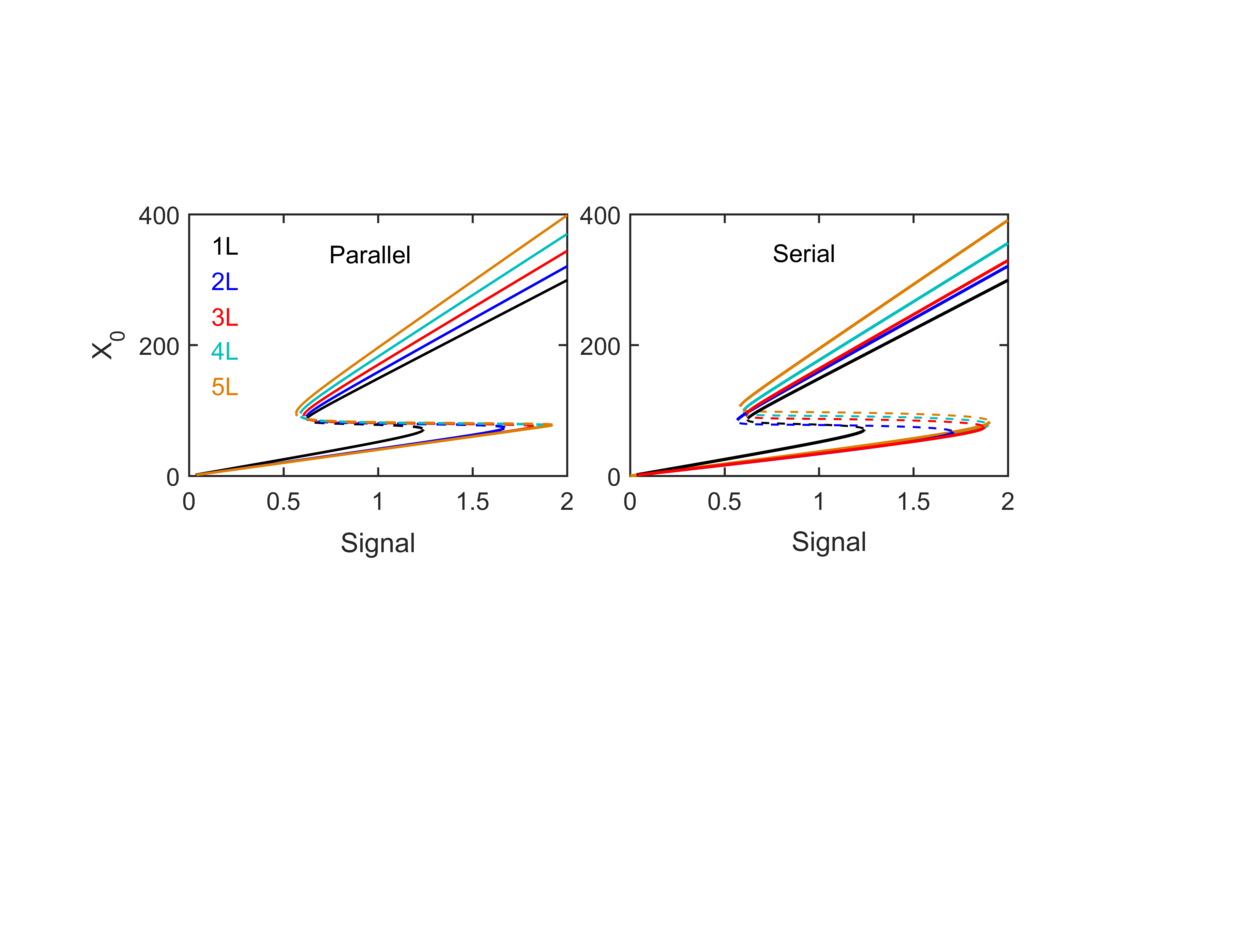

Supplement: S3 Fig — One parameter bifurcation diagrams for parallel (left) and serial (right) models with AND-gate for various number of loops with high nonlinearity (KM = 0.01) for the Goldbeter-Koshland switches. (TIF) [file pone.0188623.s004.tif]

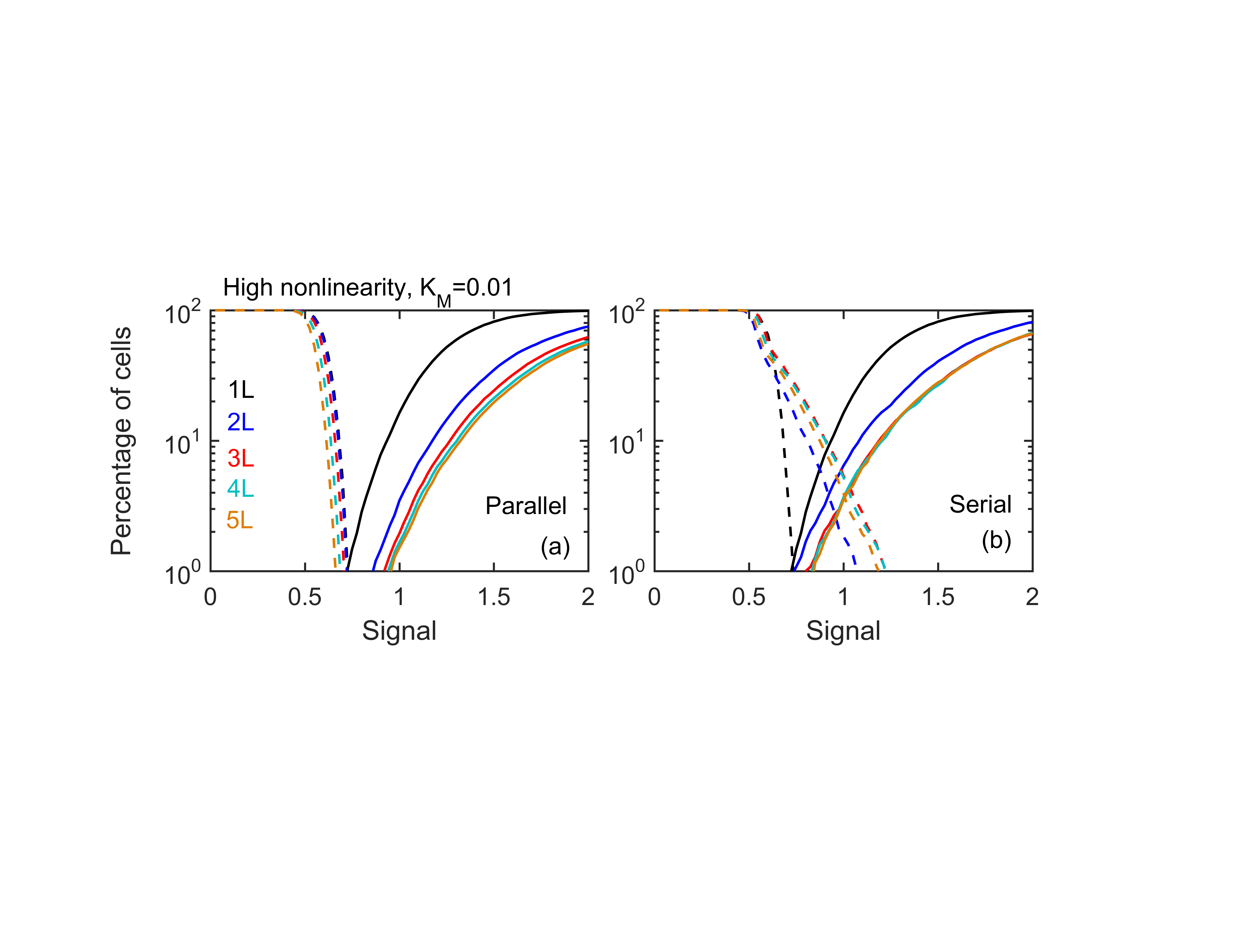

Supplement: S4 Fig — Differentiation with extrinsic noise and with high nonlinearity (KM = 0.01) for the Goldbeter-Koshland switch models with AND-gate. (a-b) The percentage of differentiated (solid) and dedifferentiated (dashed) cells with varying signal doses for parallel (left) and serial (right) regulatory motifs with different numbers of PFLs. (TIF) [file pone.0188623.s005.tif]

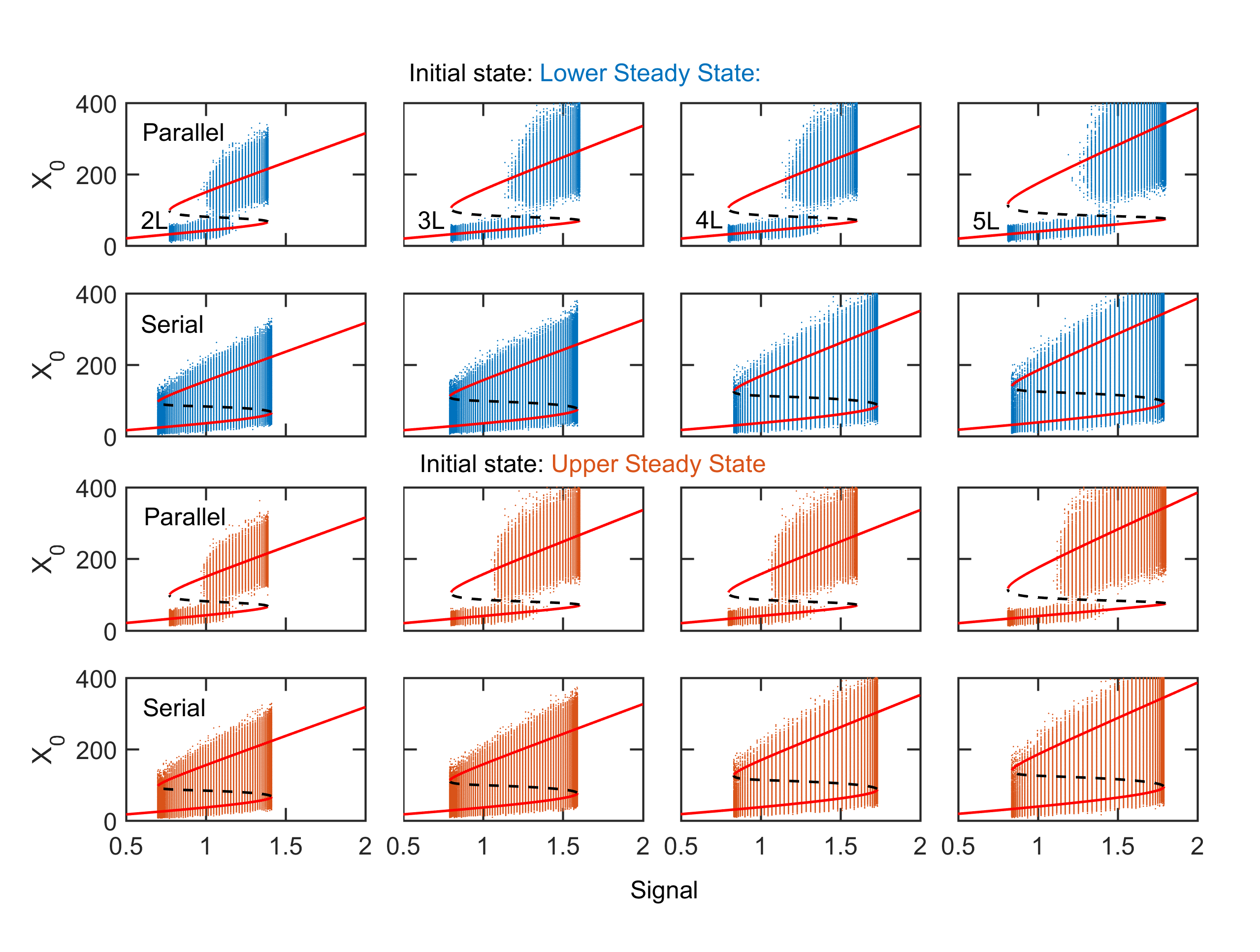

Supplement: S5 Fig — The steady state distribution of cells in the bistable region with the intrinsic noise for various number of PFLs with low nonlinearity (KM = 0.05) for the Goldbeter-Koshland switches with AND-gate. Each point here represents a cell. The upper two rows (blue) and the lower two rows (orange) have cells initialized in the lower and upper steady states respectively. (TIF) [file pone.0188623.s006.tif]

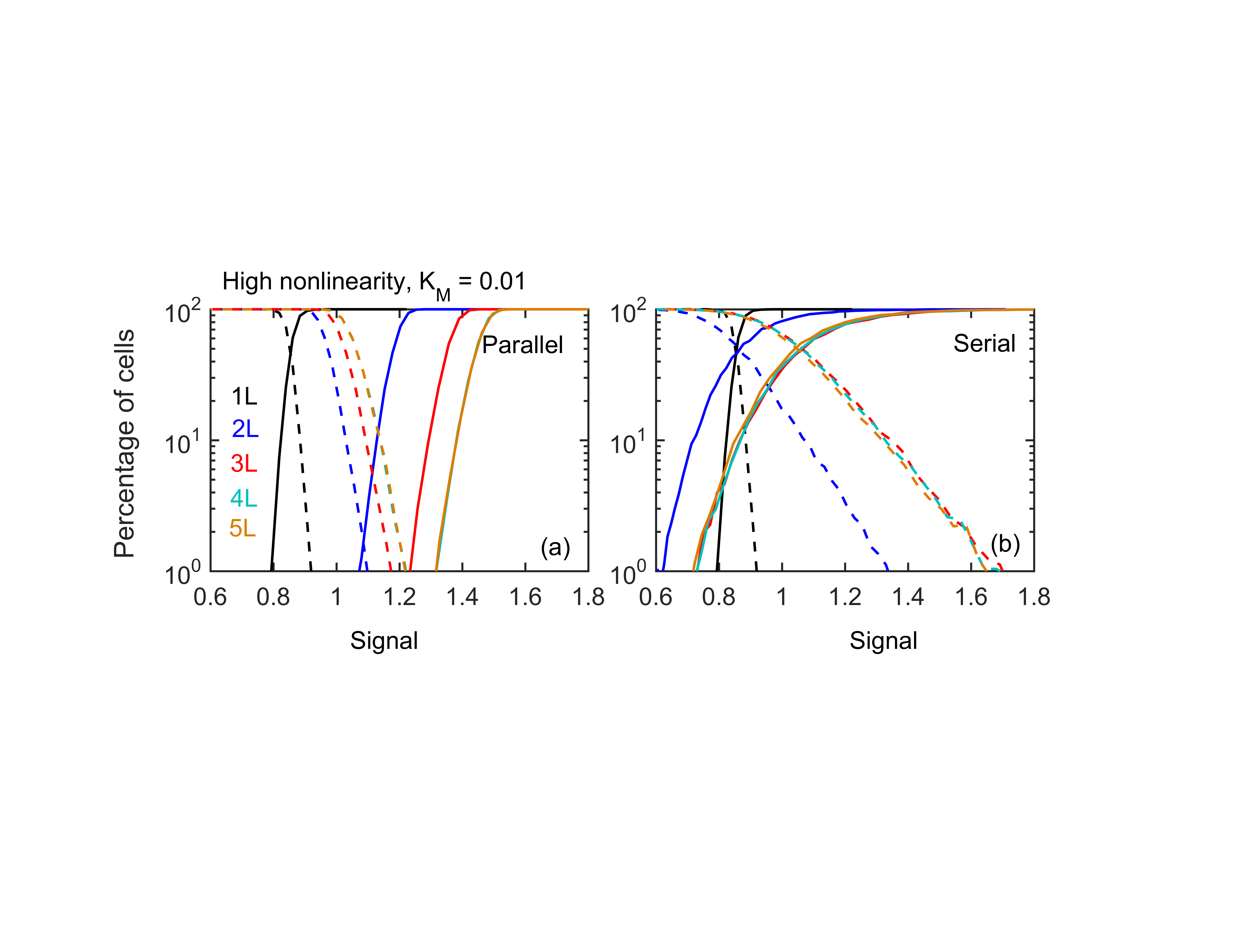

Supplement: S6 Fig — Differentiation under intrinsic noise with high nonlinearity (KM = 0.01) for the Goldbeter-Koshland switch models with AND-gate. (a-b) The percentage of differentiated (solid) and dedifferentiated (dashed) cells with varying signal doses for parallel (left) and serial (right) regulatory motifs with different numbers of PFLs are shown. (TIF) [file pone.0188623.s007.tif]

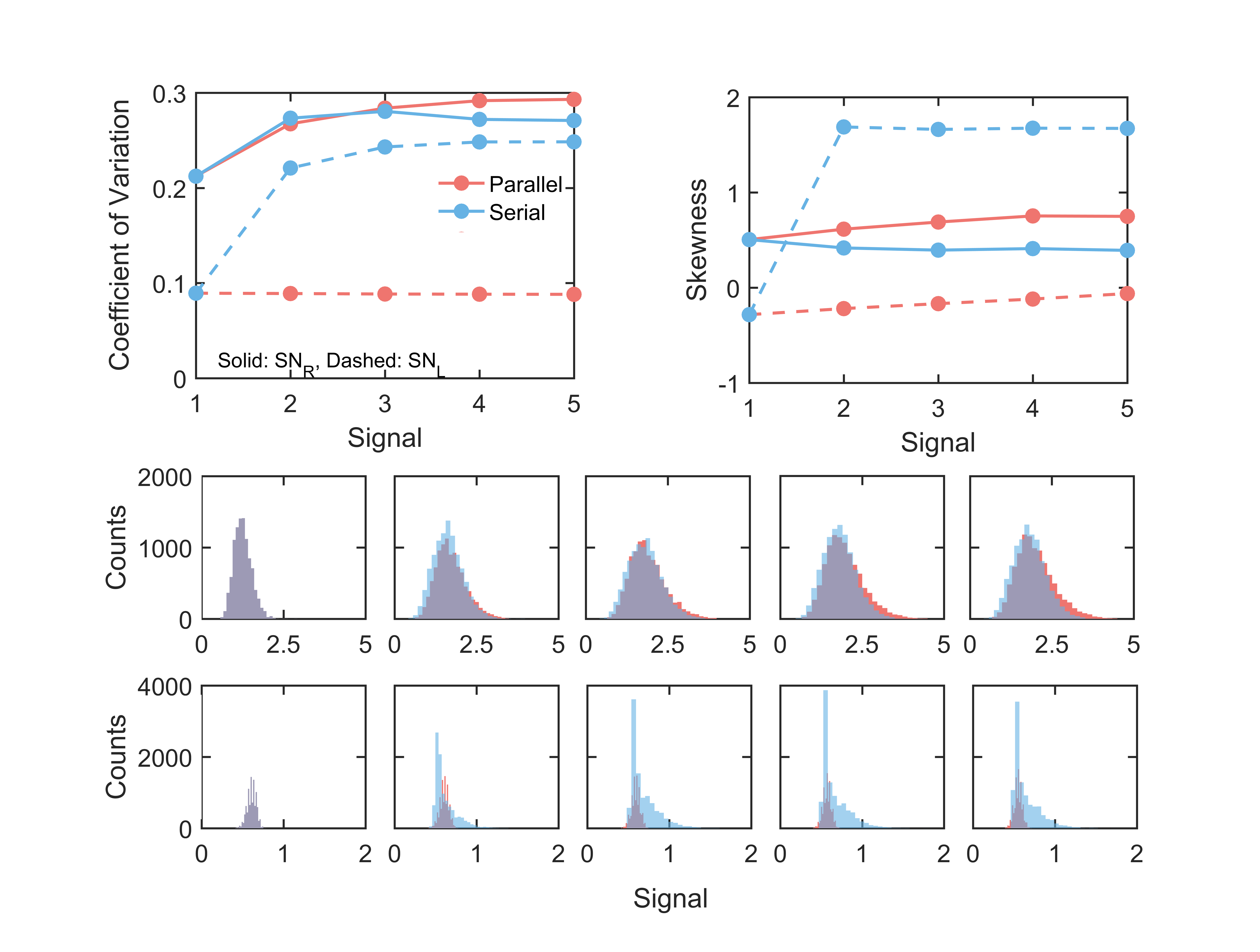

Supplement: S7 Fig — Susceptibility of bifurcation points to the extrinsic noise for the Goldbeter-Koshland switch models with AND-gate having high nonlinearity (KM = 0.01). The coefficient of variation (top left) and skewness (top right) of right (SNR) and left (SNL) bifurcation points with increasing number of PFLs are shown. Comparison of the distributions of right (top row) and left bifurcation (bottom row) points for parallel and serial models. (TIF) [file pone.0188623.s008.tif]

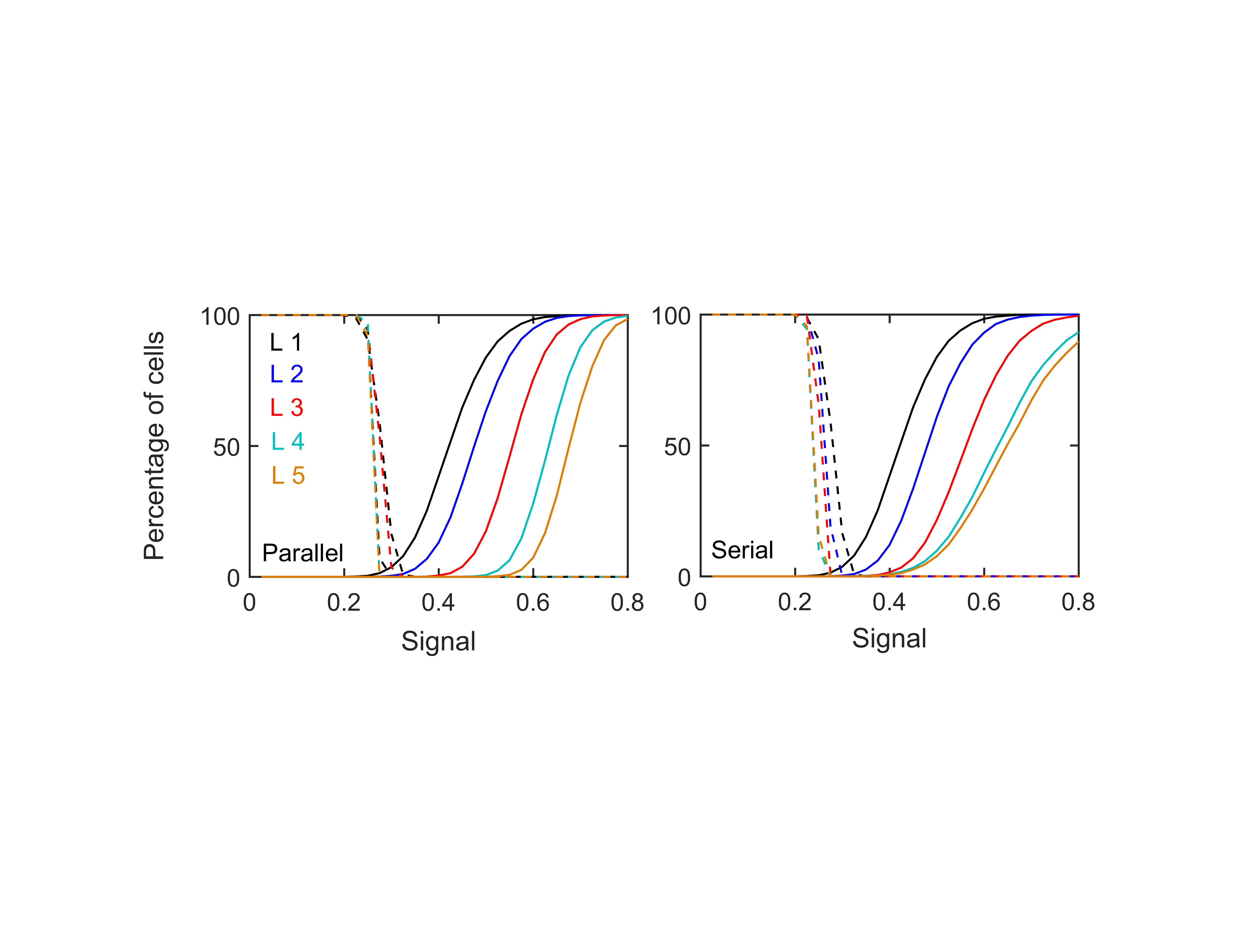

Supplement: S8 Fig — Differentiation with extrinsic noise with low nonlinearity (KM = 0.05) for the Goldbeter-Koshland switch models with OR-gate. The percentage of differentiated (solid) and dedifferentiated (dashed) cells with varying signal doses for parallel (left) and serial (right) regulatory motifs with different numbers of PFLs. (TIF) [file pone.0188623.s009.tif]

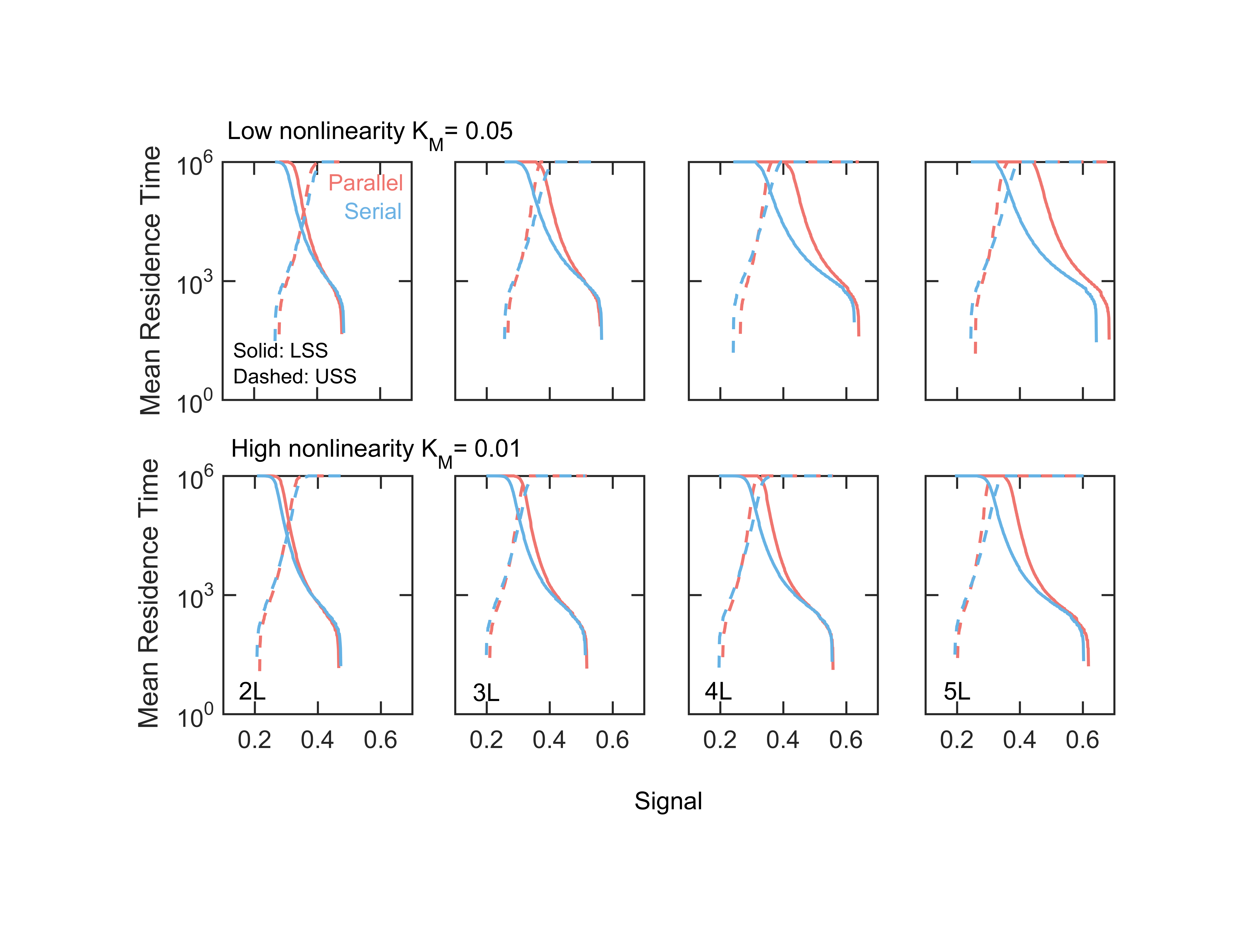

Supplement: S9 Fig — Stability of steady states under intrinsic noise: mean residence time steady states with OR-gate. Comparison of mean residence times of upper (USS) and lower (LSS) steady states for parallel and serial models with low nonlinearity (top; KM = 0.05) and High nonlinearity (bottom; KM = 0.01). The maximum simulation time was 1×10^6^ arbitrary time unit. (TIF) [file pone.0188623.s010.tif]

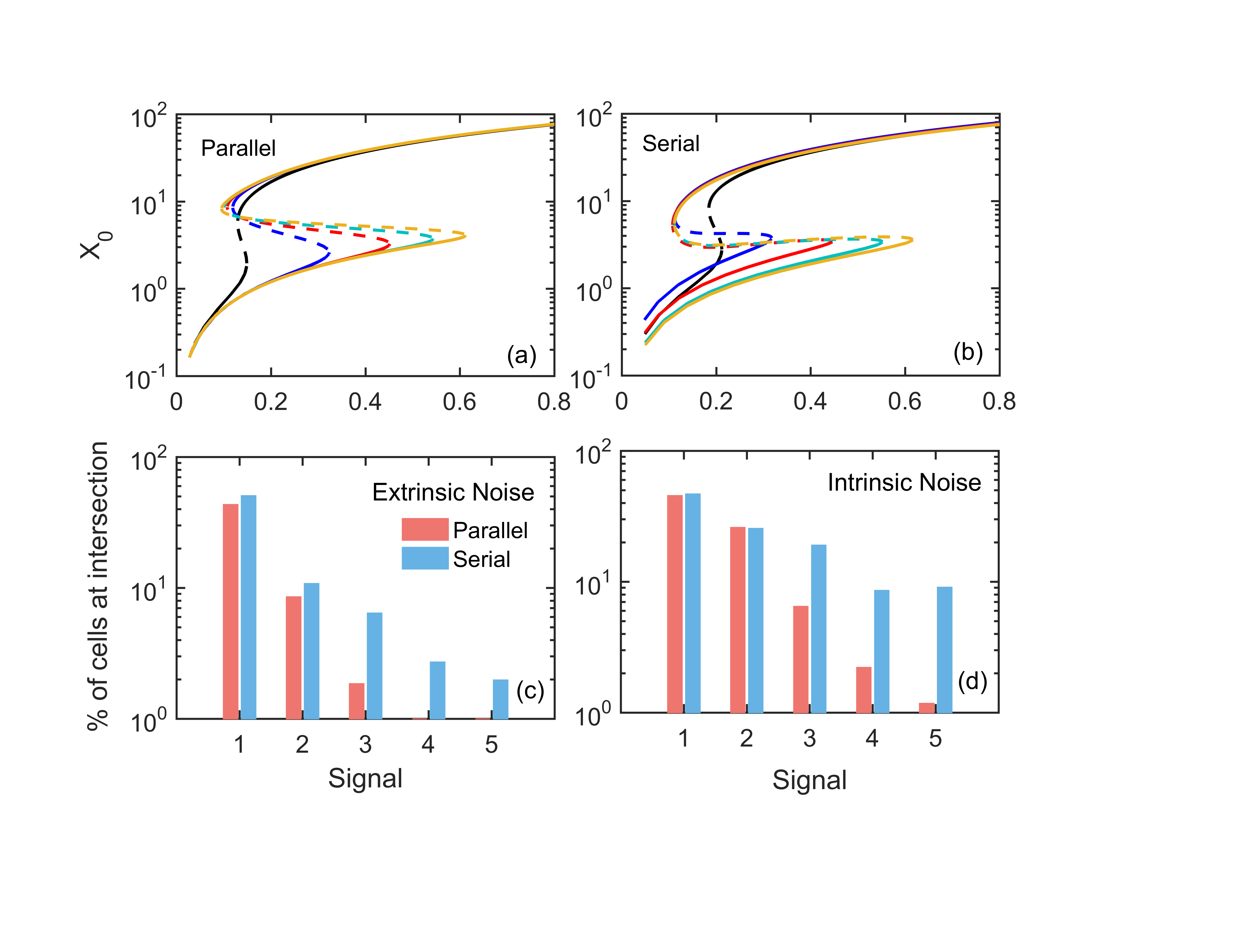

Supplement: S10 Fig — One parameter bifurcation diagrams (a-b) for parallel (left) and serial (right) models with Hill function with AND-gate for various number of loops with cooperativity (M = 2). The color scheme of lines are same as S8 Fig. Comparison of the percentage of cell at the intersection of differentiation and dedifferentiation curves for two models with various number of PFLs. (c) extrinsic noise and (d) intrinsic noise. (TIF) [file pone.0188623.s011.tif]
