# Supplementary material for: Parallel arrangements of positive feedback loops limit cell-to-cell variability in differentiation
Source: PLoS One. 2017 Nov 29;12(11):e0188623. doi: 10.1371/journal.pone.0188623 (PMC5706692; doi:10.1371/journal.pone.0188623)
Supplement: S1 Table — Parameter values for the models in AND-gate configurations. With changing number of feedback loops, the parameters whose values were adjusted to obtain similar region bistability are highlighted. Red-coloured fonts indicate that in case of extrinsic noise calculations these rate constants were sampled from independent log-normal distributions (CV = 0.3) with average value indicated in the table. The unit of rf,i becomes min-1 for i = N. The value of scaling factor (V) was 40. (DOCX) [file pone.0188623.s012.docx]

**S1 Table:** **Parameter values for the models in AND-gate configurations.** Parameter values for the models in **AND**-gate configurations. With changing number of feedback loops, the parameters whose values were adjusted to obtain similar region bistability are highlighted. Red-coloured fonts indicate that in case of extrinsic noise calculations these rate constants were sampled from independent log-normal distributions (CV=0.3) with average value indicated in the table. The unit of $r_{f,i}$ becomes ${min}^{-1}$ for $i=N$. The value of scaling factor (*V*) was 40.

| Parameters | Goldbeter-Koshland Switch: **Parallel**, **AND**-gate | | | | | | | | | | |
| --- | --- | --- | --- | --- | --- | --- | --- | --- | --- | --- | --- |
|  | Low Nonlinearity | | | | |  | High Nonlinearity | | | | |
|  | 1L | 2L | 3L | 4L | 5L |  | 1L | 2L | 3L | 4L | 5L |
| $k_{0} ({min}^{-1})$ | 0.01 | 0.01 | 0.01 | 0.01 | 0.01 |  | 0.01 | 0.01 | 0.01 | 0.01 | 0.01 |
| $k_{1}$  $\left( molecule^{-N}{min}^{-1} \right)$ | 0.025 | 0.025 | 0.025 | 0.025 | 0.025 |  | 0.025 | 0.025 | 0.025 | 0.025 | 0.025 |
| $\gamma\left( {min}^{-1} \right)$ | 0.01 | 0.01 | 0.01 | 0.01 | 0.01 |  | 0.01 | 0.01 | 0.01 | 0.01 | 0.01 |
| $k_{2}^{'} \left( {min}^{-1} \right)$ | 0.01 | 0.01 | 0.01 | 0.01 | 0.01 |  | 0.01 | 0.01 | 0.01 | 0.01 | 0.01 |
| $T_{T} (molecule)$ | 1 | 1 | 1 | 1 | 1 |  | 1 | 1 | 1 | 1 | 1 |
| $k_{2} (molecule {min}^{-1})$ | 0.001 | 0.001 | 0.001 | 0.001 | 0.001 |  | 0.001 | 0.001 | 0.001 | 0.001 | 0.001 |
| $k_{f} \left( {min}^{-1} \right)$ | 0.1 | 0.1 | 0.1 | 0.1 | 0.1 |  | 0.1 | 0.1 | 0.1 | 0.1 | 0.1 |
| $k_{b} (molecule {min}^{-1})$ | 0.2 | 0.2 | 0.2 | 0.2 | 0.2 |  | 0.2 | 0.2 | 0.2 | 0.2 | 0.2 |
| $K_{M} (molecule)$ | 0.05 | 0.05 | 0.05 | 0.05 | 0.05 |  | 0.01 | 0.01 | 0.01 | 0.01 | 0.01 |
|  | Goldbeter-Koshland Switch: **Serial**, **AND**-gate | | | | | | | | | | |
| $r_{0} ({min}^{-1})$ | 0.01 | 0.005 | 0.005 | 0.005 | 0.005 |  | 0.01 | 0.005 | 0.005 | 0.005 | 0.005 |
| $r_{1}$  $\left( molecule^{-1}{min}^{-1} \right)$ | 0.025 | 0.032 | 0.033 | 0.036 | 0.04 |  | 0.025 | 0.032 | 0.033 | 0.036 | 0.04 |
| $\gamma\left( {min}^{-1} \right)$ | 0.01 | 0.01 | 0.01 | 0.01 | 0.01 |  | 0.01 | 0.01 | 0.01 | 0.01 | 0.01 |
| $r_{2}^{'} \left( {min}^{-1} \right)$ | 0.01 | 0.01 | 0.01 | 0.01 | 0.01 |  | 0.01 | 0.01 | 0.01 | 0.01 | 0.01 |
| $T_{T} (molecule)$ | 1 | 1 | 1 | 1 | 1 |  | 1 | 1 | 1 | 1 | 1 |
| $r_{2} (molecule {min}^{-1})$ | 0.001 | 0.001 | 0.001 | 0.001 | 0.001 |  | 0.001 | 0.001 | 0.001 | 0.001 | 0.001 |
| $r_{f,1}$ $\left( {min}^{-1} \right)$ | 0.1 | 0.85 | 0.75 | 0.65 | 0.59 |  | 0.1 | 1.0 | 0.9 | 0.85 | 0.8 |
| $r_{f,2}$  $\left( molecule^{-1}{min}^{-1} \right)$ | 0.06 | 0.06 | 0.06 | 0.06 | 0.06 |  | 0.06 | 0.06 | 0.06 | 0.06 | 0.06 |
| $r_{f,i} \left( i=3,4,5 \right)$  $\left( molecule^{-1}{min}^{-1} \right)$ | 2.0 | 2.0 | 2.0 | 2.0 | 2.0 |  | 2.0 | 2.0 | 2.0 | 2.0 | 2.0 |
| $r_{b} (molecule {min}^{-1})$ | 0.2 | 0.2 | 0.2 | 0.2 | 0.2 |  | 0.2 | 0.2 | 0.2 | 0.2 | 0.2 |
| $K_{M} (molecule)$ | 0.05 | 0.05 | 0.05 | 0.05 | 0.05 |  | 0.01 | 0.01 | 0.01 | 0.01 | 0.01 |
