# Supplementary material for: Parallel arrangements of positive feedback loops limit cell-to-cell variability in differentiation
Source: PLoS One. 2017 Nov 29;12(11):e0188623. doi: 10.1371/journal.pone.0188623 (PMC5706692; doi:10.1371/journal.pone.0188623)
Supplement: S2 Table — List of chemical reactions and their propensities in parallel and serial models with GK switch. (DOCX) [file pone.0188623.s013.docx]

**S2 Table. List of chemical reactions and their propensities.** List of chemical reactions and their propensities in parallel and serial models with GK switch.

| **Reaction**  **Number** | **Chemical**  **Reactions** | **Propensities** | |
| --- | --- | --- | --- |
|  |  | **Parallel** | **Serial** |
| 1. | $X_{0}$ | $s\left( k_{0}V+ k_{1}\mathcal{P}_{0} \right)$ | $s\left( r_{0}V+r_{1}X_{1} \right)$ |
| 2. | $X_{0}$ | $\gamma X_{0}$ | $\gamma X_{0}$ |
| 3. | $X_{i}$ | $k_{2}V+k_{2}^{'}T_{i,A}$ | $r_{2}V+r_{2}^{'}T_{i,A}$ |
| 4. | $X_{i}$ | $\gamma X_{i}$ | $\gamma X_{i}$ |
| 5. | $T_{i,I}$ $T_{i,A}$ | $\frac{k_{f}\left( V.T_{T}-T_{i,A} \right)X_{0}}{K_{M}V+\left( V.T_{T}-T_{i,A} \right)}$ | $\frac{r_{f}\left( V.T_{T}-T_{i,A} \right)\mathcal{G}_{i}}{K_{M}V+\left( V.T_{T}-T_{i,A} \right)}$ |
| 6. | $T_{i,A}$ $T_{i,I}$ | $\frac{k_{b}V.T_{i,A}}{K_{M}V+T_{i,A}}$ | $\frac{r_{b}V.T_{i,A}}{K_{M}V+T_{i,A}}$ |
| $N$ is the number of positive feedback loops  **Parallel Motifs:** For AND-gate $\mathcal{P}_{0}=\frac{1}{V^{N-1}}\prod_{i=1}^{N} X_{i}$ and for OR-gate $\mathcal{P}_{0}= \sum_{i=1}^{N} X_{i}$  **Serial Motifs:** For AND-gate $\mathcal{G}_{i}={\frac{1}{V}X}_{i-1}X_{i+1}$ and for OR-gate $\mathcal{G}_{i}=X_{i-1}{+X}_{i+1}$; for $N=1, \mathcal{G}_{1}=X_{0}$ and for $i=N, \mathcal{G}_{N}=X_{N-1}$ | | | |
