# Supplementary material for: Parallel arrangements of positive feedback loops limit cell-to-cell variability in differentiation
Source: PLoS One. 2017 Nov 29;12(11):e0188623. doi: 10.1371/journal.pone.0188623 (PMC5706692; doi:10.1371/journal.pone.0188623)
Supplement: S3 Table — Parameter values for the models in OR-gate configurations. With changing number of feedback loops, the parameters whose values were adjusted to obtain similar region bistability are highlighted. Red-coloured fonts indicate that in case of extrinsic noise calculations these rate constants were sampled from independent log-normal distributions (CV = 0.3) with average value indicated in the table. The value of scaling factor (V) was 40. (DOCX) [file pone.0188623.s014.docx]

**S3 Table. Parameter values for the models in OR-gate configurations.** Parameter values for the models in **OR**-gate configurations. With changing number of feedback loops, the parameters whose values were adjusted to obtain similar region bistability are highlighted. Red-coloured fonts indicate that in case of extrinsic noise calculations these rate constants were sampled from independent log-normal distributions (CV=0.3) with average value indicated in the table. The value of scaling factor (*V*) was 40.

| Parameters | Goldbeter-Koshland Switch: **Parallel**, **OR**-gate | | | | | | | | | | |
| --- | --- | --- | --- | --- | --- | --- | --- | --- | --- | --- | --- |
|  | Low Nonlinearity | | | | |  | High Nonlinearity | | | | |
|  | 1L | 2L | 3L | 4L | 5L |  | 1L | 2L | 3L | 4L | 5L |
| $k_{0} ({min}^{-1})$ | 0.02 | 0.014 | 0.0071 | 0.003 | 0.001 |  | 0.03 | 0.025 | 0.02 | 0.017 | 0.013 |
| $k_{1}$  $\left( molecule^{-1}{min}^{-1} \right)$ | 0.07 | 0.039 | 0.03 | 0.024 | 0.02 |  | 0.07 | 0.038 | 0.028 | 0.022 | 0.019 |
| $\gamma\left( {min}^{-1} \right)$ | 0.01 | 0.01 | 0.01 | 0.01 | 0.01 |  | 0.01 | 0.01 | 0.01 | 0.01 | 0.01 |
| $k_{2}^{'} \left( {min}^{-1} \right)$ | 0.01 | 0.01 | 0.01 | 0.01 | 0.01 |  | 0.01 | 0.01 | 0.01 | 0.01 | 0.01 |
| $T_{T} (molecule)$ | 1 | 1 | 1 | 1 | 1 |  | 1 | 1 | 1 | 1 | 1 |
| $k_{2} (molecule {min}^{-1})$ | 0.001 | 0.001 | 0.001 | 0.001 | 0.001 |  | 0.001 | 0.001 | 0.001 | 0.001 | 0.001 |
| $k_{f} \left( {min}^{-1} \right)$ | 0.1 | 0.1 | 0.1 | 0.1 | 0.1 |  | 0.1 | 0.1 | 0.1 | 0.1 | 0.1 |
| $k_{b} (molecule {min}^{-1})$ | 0.2 | 0.2 | 0.2 | 0.2 | 0.2 |  | 0.2 | 0.2 | 0.2 | 0.2 | 0.2 |
| $K_{M} (molecule)$ | 0.05 | 0.05 | 0.05 | 0.05 | 0.05 |  | 0.01 | 0.01 | 0.01 | 0.01 | 0.01 |
|  | Goldbeter-Koshland Switch: **Serial**, **OR** gate | | | | | | | | | | |
| $r_{0} ({min}^{-1})$ | 0.02 | 0.011 | 0.005 | 0.001 | 5×10^-4^ |  | 0.03 | 0.022 | 0.018 | 0.015 | 0.012 |
| $r_{1}$  $\left( molecule^{-1}{min}^{-1} \right)$ | 0.07 | 0.08 | 0.09 | 0.1 | 0.1 |  | 0.07 | 0.078 | 0.086 | 0.09 | 0.095 |
| $\gamma\left( {min}^{-1} \right)$ | 0.01 | 0.01 | 0.01 | 0.01 | 0.01 |  | 0.01 | 0.01 | 0.01 | 0.01 | 0.01 |
| $r_{2}^{'} \left( {min}^{-1} \right)$ | 0.01 | 0.01 | 0.01 | 0.01 | 0.01 |  | 0.01 | 0.01 | 0.01 | 0.01 | 0.01 |
| $T_{T} (molecule)$ | 1 | 1 | 1 | 1 | 1 |  | 1 | 1 | 1 | 1 | 1 |
| $r_{2} (molecule {min}^{-1})$ | 0.001 | 0.001 | 0.001 | 0.001 | 0.001 |  | 0.001 | 0.001 | 0.001 | 0.001 | 0.001 |
| $r_{f,i} \left( {min}^{-1} \right)$ | 0.1 | 0.1 | 0.1 | 0.1 | 0.1 |  | 0.1 | 0.1 | 0.1 | 0.1 | 0.1 |
| $r_{b}(molecule {min}^{-1})$ | 0.2 | 0.2 | 0.2 | 0.2 | 0.2 |  | 0.2 | 0.2 | 0.2 | 0.2 | 0.2 |
| $K_{M} (molecule)$ | 0.05 | 0.05 | 0.05 | 0.05 | 0.05 |  | 0.01 | 0.01 | 0.01 | 0.01 | 0.01 |
