# Supplementary material for: Parallel arrangements of positive feedback loops limit cell-to-cell variability in differentiation
Source: PLoS One. 2017 Nov 29;12(11):e0188623. doi: 10.1371/journal.pone.0188623 (PMC5706692; doi:10.1371/journal.pone.0188623)
Supplement: S4 Table — List of dynamical equations for models with Hill function. (DOCX) [file pone.0188623.s015.docx]

**S4 Table. Dynamical equations for the models with Hill function.** List of dynamical equations for models with Hill function.

| **Parallel** | **Serial** |
| --- | --- |
| $\frac{dX_{0}}{dt}= \varepsilon_{0}s\left( V+\frac{\alpha V \prod_{i}^{N} X_{i}^{M}}{V^{NM}+ \prod_{i}^{N} X_{i}^{M}} \right)- \gamma X_{0}$ | $\frac{dX_{0}}{dt}= s\left( k_{0}V+p_{0}X_{1} \right)- \gamma X_{0}$ |
| $\frac{dX_{i}}{dt}= \varepsilon_{i}X_{0}- \gamma X_{i}$ | $\frac{dX_{i}}{dt}=k_{i}V+ \frac{p_{i}V X_{i-1}^{M} X_{i+1}^{M}}{b_{1}V^{2M}+ X_{i-1}^{M} X_{i+1}^{M}}- \gamma X_{i}$ |
| $N$is the number of PFLs and *M* is the cooperativity, $X_{N+1}\boldsymbol{=}1$ | |
