# Supplementary material for: Parallel arrangements of positive feedback loops limit cell-to-cell variability in differentiation
Source: PLoS One. 2017 Nov 29;12(11):e0188623. doi: 10.1371/journal.pone.0188623 (PMC5706692; doi:10.1371/journal.pone.0188623)
Supplement: S5 Table — Parameters values for the models with Hill function. Red-coloured fonts indicate that in case of extrinsic noise calculations these rate constants were sampled from independent log-normal distributions (CV = 0.3) with average value indicated in the table. The value of scaling factor (V) was 30. (DOCX) [file pone.0188623.s016.docx]

**S5 Table. Parameters values for the models with Hill function.** Parameters values for the models with Hill function. Red-coloured fonts indicate that in case of extrinsic noise calculations these rate constants were sampled from independent log-normal distributions (CV=0.3) with average value indicated in the table. The value of scaling factor (*V*) was 30.

| Parameters | Hill Function: AND gate, M = 2 | | | | |
| --- | --- | --- | --- | --- | --- |
|  | **Parallel** | | | | |
|  | 1L | 2L | 3L | 4L | 5L |
| $\varepsilon_{0} ({min}^{-1})$ | 0.002 | 0.002 | 0.002 | 0.002 | 0.002 |
| $\varepsilon_{i} ({min}^{-1})$ | 0.045 | 0.045 | 0.045 | 0.045 | 0.045 |
| α | 15 | 15 | 15 | 15 | 15 |
| $\gamma\left( {min}^{-1} \right)$ | 0.01 | 0.01 | 0.01 | 0.01 | 0.01 |
|  | **Serial** | | | | |
| $k_{0} ({min}^{-1})$ | 0.001 | 0.001 | 0.001 | 0.001 | 0.001 |
| $k_{1} (molecule {min}^{-1})$ | 0.001 | 0.002 | 0.0011 | 0.0006 | 0.0005 |
| $k_{i}\left( i=2,3,4,5 \right)$  $(molecule {min}^{-1})$ | - | 0.005 | 0.005 | 0.005 | 0.005 |
| $p_{0} (molecule^{-1} {min}^{-1})$ | 0.01 | 0.01 | 0.01 | 0.01 | 0.01 |
| $p_{1} (molecule {min}^{-1})$ | 0.03 | 0.03 | 0.03 | 0.03 | 0.03 |
| $p_{2} (molecule {min}^{-1})$ | - | 0.035 | 0.035 | 0.035 | 0.035 |
| $p_{3} (molecule {min}^{-1})$ | - | - | 0.015 | 0.015 | 0.015 |
| $p_{4} (molecule {min}^{-1})$ | - | - | - | 0.05 | 0.05 |
| $p_{5} (molecule {min}^{-1})$ | - | - | - | - | 0.05 |
| $b_{1} \left( {molecule}^{2M} \right)$ | 0.1 | 0.5 | 0.5 | 0.5 | 0.5 |
| $\gamma\left( {min}^{-1} \right)$ | 0.01 | 0.01 | 0.01 | 0.01 | 0.01 |
